# Evaluation of the Antifungal Properties of Azomethine‐Pyrazole Derivatives from a Structural Perspective

**DOI:** 10.1002/open.202500132

**Published:** 2025-04-28

**Authors:** María Isabel Murillo, Andrés Camilo Restrepo‐Acevedo, Cristian Rocha‐Roa, Susana Zacchino, Laura Svetaz, Simón Hernández‐Ortega, Rodrigo Abonia, Ronan Le Lagadec, Fernando Cuenú‐Cabezas

**Affiliations:** ^1^ Laboratorio de Química inorgánica y catálisis Programa de Química Universidad del Quindío Carrera 15, Calle 12 Norte Armenia Colombia; ^2^ Instituto de Química UNAM Circuito Exterior s/n, Ciudad Universitaria 04510 Ciudad de México, México; ^3^ Grupo GEPAMOL Centro de Investigaciones Biomédicas Universidad del Quindío Carrera 15, Calle 12 Norte Armenia 630004 Colombia; ^4^ Department of Biology University of Fribourg Fribourg CH 1700 Switzerland; ^5^ Área Farmacognosia Facultad de Ciencias Bioquímicas y Farmacéuticas Universidad Nacional de Rosario Suipacha 531 2000 Rosario Argentina; ^6^ Departamento de Química Universidad del Valle Calle 13 No. 100–00, A.A. 25360 Cali Colombia

**Keywords:** Pyrazoles, Azomethines, Antifungal activity, Candida, DFT and molecular docking calculations

## Abstract

About 95 % of candidiasis infections worldwide are attributed to five *Candida* fungi species, with *C. albicans* being the most prevalent and severe. Due to resistance phenomena, the last decade has seen a significant challenge for candidiasis treatment with antifungal drugs, which has led to an urgent need for new antifungal agents. In this article, we report the synthesis of a series of azomethine‐pyrazole derivatives bearing a *para*‐substituted azo‐phenyl ring. These compounds were evaluated as antifungal agents against *Candida* species and *Cryptococcus neoformans* strains. Compound **ClAzoNH**, substituted by chloride, displayed the highest toxicity on *Candida albicans*, with an MIC_50_ value of 2.08 μg/mL, while methoxy‐substituted **MeOAzoNH** showed moderate inhibitory activity. The unsubstituted **AzoNH** compound exhibited the highest activity towards *Candida tropicalis*, *Candida glabrata*, *Candida parapsilosis*, and *Candida krusei* strains. In the case of *C. albicans*, the *Ca*CYP51 protein appears to be the most probable biological target, while for *C. neoformans*, interactions with the *Cn*FTase protein explained the *in vitro* results.

## Graphical abstract

A series of azomethine‐pyrazole derivatives bearing a *para*‐substituted azo‐phenyl ring is straightforwardly prepared. The new compounds display an elevated antifungal activity against various *Candida* species and *Cryptococcus neoformans* strains. DFT and molecular docking calculations are used to determine possible routes of action through interactions with the *Ca*CYP51 and *Cn*FTase proteins.

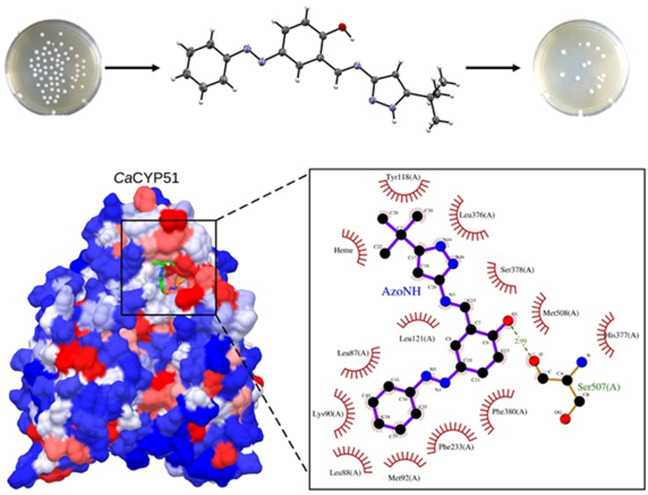

## Introduction

**Figure 1:**
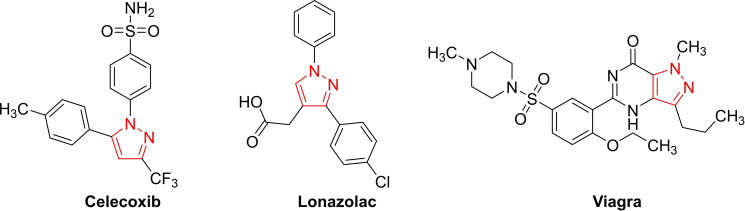
Commercial drugs containing pyrazole moieties.

Pyrazoles, five‐membered heterocycles containing two adjacent nitrogen atoms, are core structures found in numerous pharmaceutically active molecules (Figure 1).^[1]^ For instance, nonsteroidal anti‐inflammatory drugs (NSAID), Celecoxib, and Lonozalac are di‐aryl pyrazole derivatives.^[2]^ Other pyrazole‐based compounds have displayed activities such as antihypertensive (Viagra),^[3]^ antidepressant,^[4]^ anti‐inflammatory,^[5]^ antimicrobial,^[6]^ analgesic,^[7]^ anticancer,^[8]^ anticonvulsant,^[9]^ antiobesity,^[10]^ antiviral,^[11]^ hypoglycemic,^[12]^ leishmanicidal,^[13]^ and antituberculosis.^[14]^

**Figure 2:**
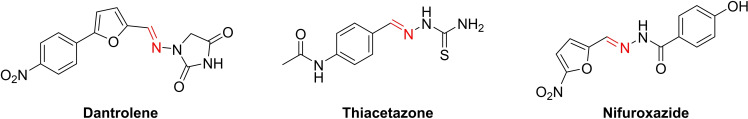
Commercial drugs containing Schiff base moieties.

On the other hand, Schiff bases are formed from an aldehyde and a primary amine, giving rise to an unsaturated imine or azomethine group (−CH=N−).[^15^, ^16^, ^17^] Schiff bases have exhibited a wide range of biological activity, as they are present in the structure of several pharmacophores, such as Dandrolene used to treat malignant hyperthermia, Thiacetazone used as an antibiotic, and Nifuroxazide used as an intestinal antimicrobial (Figure 2). Aromatic Schiff bases have also been applied as effective herbicides, pesticides, and bactericides.[^18^, ^19^]

Additionally, azo compounds present a typical −N=N− double bond and a more extensive π‐electron conjugation system, which allows a wide range of applications as functional materials. In addition, heteroaryl‐based azo dyes have been studied for biological applications as antioxidant,^[20]^ antimicrobial,^[21]^ antitumor,^[22]^ antidiabetic,^[23]^ and antiviral^[24]^ agents.

Candidiasis are mycosis caused by various species of opportunistic yeasts of the genus *Candida spp*. They are found in the human mouth, skin, and gastrointestinal tract microflora, and the innate immune system usually regulates their overgrowth.^[25]^ However, many *Candida spp*. can acquire pathogenic properties and cause various diseases ranging from superficial skin, hair, and nail infections to life‐threatening systemic and blood infections.^[26]^ Worldwide, approximately 95 % of candidiasis infections are attributed to 5 *Candida* species (*C. albicans, C. glabrata, C. parapsilosis, C. tropicalis*, and *C. krusei*), with *C. albicans* being the most prevalent and most severe.^[27]^

The current increase in antifungal drug resistance and clinical treatment failure of *Candida spp*. is of concern because invasive candidiasis represents a significant cause of mortality in intensive care units. This problem has led to the design of new broad‐spectrum antifungal agents such as triazoles and echinocandins. However, *Candida spp* is developing resistance to these drugs, highlighting the challenge of expanding the spectrum of antifungal agents.^[28]^

**Figure 3:**
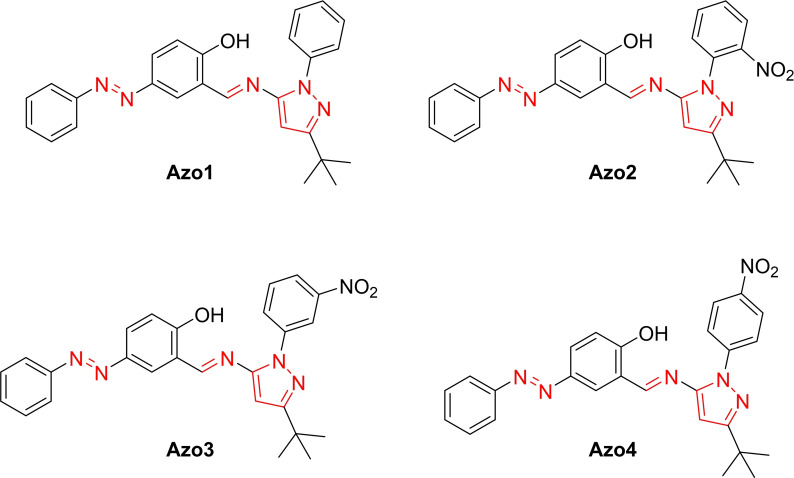
Structures of azomethine‐pyrazoles with antifungal activity.^[29]^

In previous studies, we demonstrated the antifungal activity of azomethine‐pyrazoles against certified strains of *Candida albican*s and *Cryptococcus neoformans* (Figure 3).^[29]^ The antifungal assays revealed a high to moderate inhibitory activity against both strains, which is regulated by the presence and location of the nitro group on the aromatic ring.

In the present study, new molecules bearing an *NH*‐pyrazole ring, an imine function, and an azo group were designed and synthesized. Removing the phenyl or nitrophenyl moiety on the pyrazole ring and introducing substituents on the aromatic ring of the azo fragment increased the antifungal activity. Molecular docking studies were carried out to correlate the structure‐activity relationship, and strong interactions between the new compounds and specific fungi proteins allowed to explain the *in vitro* observations.

## Results and Discussion

### Synthesis

**Scheme 1:**
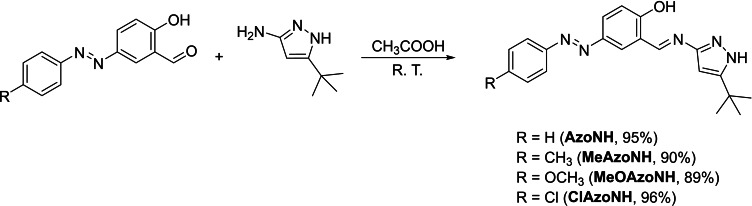
Synthesis of azomethine‐pyrazoles.

The 5‐(*tert*‐butyl)‐1*H*‐pyrazol‐3‐amine aminopyrazole was obtained following a previously reported procedure.^[30]^ The synthesis of the azo‐aldehyde precursors (*E*)‐2‐hydroxy‐5‐(phenyldiazenyl)benzaldehyde, (*E*)‐2‐hydroxy‐5‐(*p*‐tolyldiazenyl)benzaldehyde, (*E*)‐2‐hydroxy‐5‐((4‐methoxyphenyl)diazenyl)benzaldehyde and (*E*)‐5‐((4‐chlorophenyl)diazenyl)‐2‐hydroxybenzaldehyde was carried out as reported in the literature.^[31]^ The new azomethine‐pyrazoles 2‐((*E*)‐((5‐(*tert*‐butyl)‐1*H*‐pyrazol‐3‐yl)imino)methyl)‐4‐((*E*)‐phenyldiazenyl)phenol (**AzoNH**), 2‐((*E*)‐((5‐(*tert*‐butyl)‐1*H*‐pyrazol‐3‐yl)imino)methyl)‐4‐((*E*)‐*p*‐tolyldiazenyl)phenol (**MeAzoNH**), 2‐((*E*)‐((5‐(*tert*‐butyl)‐1*H*‐pyrazol‐3‐yl)imino)methyl)‐4‐((*E*)‐(4‐methoxyphenyl)diazenyl)phenol (**MeOAzoNH**), and 2‐((*E*)‐((5‐(*tert*‐butyl)‐1*H*‐pyrazol‐3‐yl)imino)methyl)‐4‐((*E*)‐(4‐chlorophenyl)diazenyl)phenol (**ClAzoNH**) were obtained from the reaction between the aminopyrazole and the corresponding azo‐aldehyde in 89 to 96 % yields (Scheme 1).[^29^, ^32^, ^33^, ^34^, ^35^] The new azomethine‐pyrazoles were characterized by IR, mass spectrometry, and NMR. The IR, mass‐, and ^1^H‐ and ^13^C‐NMR spectra are presented in Figures S1–S16.

#### X‐Ray Diffraction Crystallography

**Figure 4:**
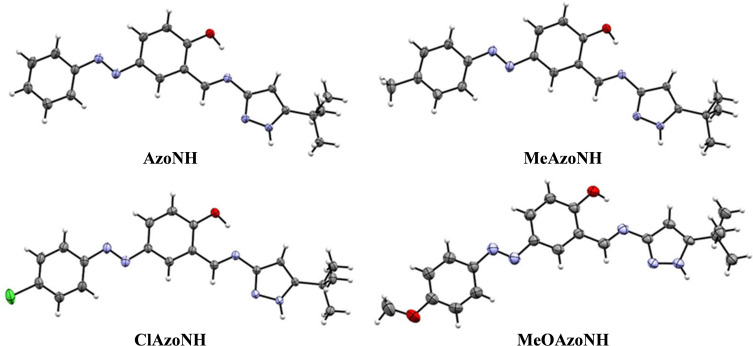
ORTEP diagrams of the asymmetric unit of compounds **AzoNH**, **MeAzoNH**, **ClAzoNH**, and **MeOAzoNH** at 50 % of probability.

Single crystals suitable for X‐ray diffraction of all four compounds were obtained by slowly evaporating dichloromethane solutions at room temperature. Figure 4 shows the ORTEP diagrams of **AzoNH**, **MeAzoNH**, **ClAzoNH**, and **MeOAzoNH**. The crystallographic data and refinement details are presented in Table S1. **AzoNH**, **MeAzoNH**, and **ClAzoNH** crystallized in the triclinic space group, while **MeOAzoNH** crystallized in the orthorhombic space group.

**Figure 5:**
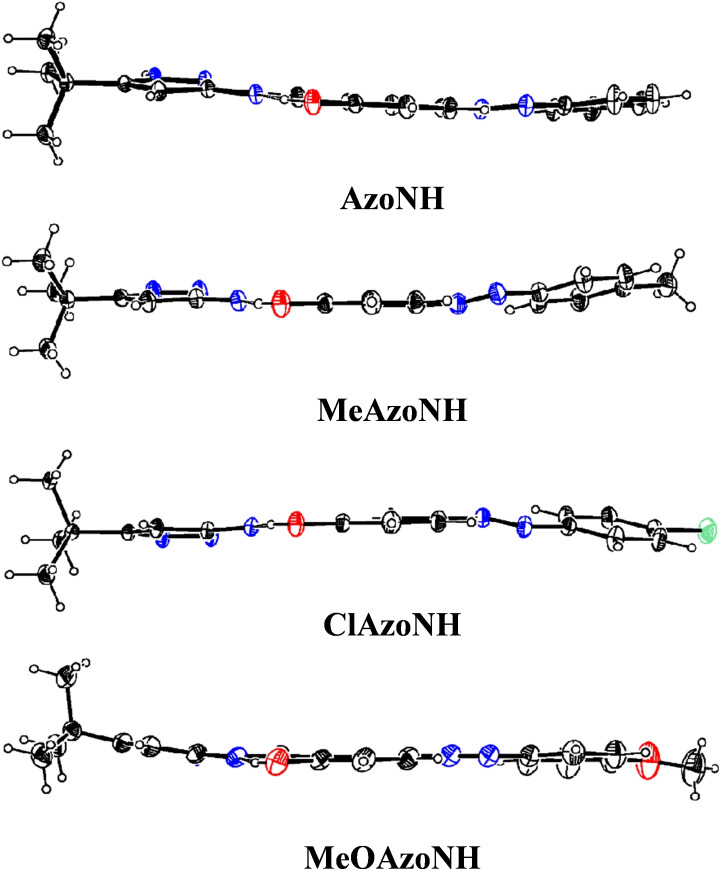
ORTEP structures of compounds **AzoNH**, **MeAzoNH**, **ClAzoNH**, and **MeOAzoNH**.

All structures are almost planar, disregarding the *tert*‐butyl group. Considering the benzylidenimine ring as reference, in **AzoNH**, the pyrazole and azophenyl rings are rotated by 8.8(1)° and 7.7(1)° respectively, while in **MeAzoNH** the dihedral angles are 7.9(2)° and 12.8(2)°, similar to those found in **ClAzoNH** (7.9(2)° and 12.4(2)°, respectively). In **MeOAzoNH**, dihedral angles of 5.9(2)° and 8.7(2)° were observed, indicating a slightly more planar structure (Figure 5).

**Figure 6:**
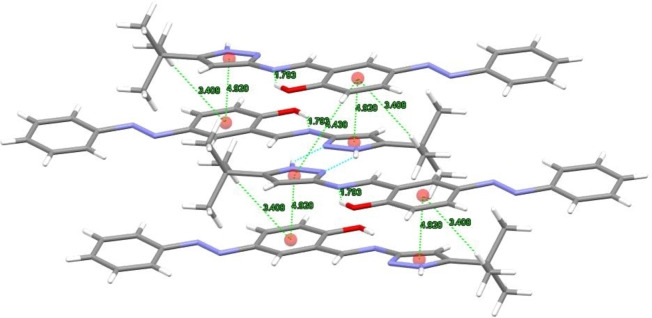
Centroid‐centroid interaction distances for **AzoNH**.

All compounds showed strong intramolecular interactions between the hydroxyl group as hydrogen bonding donor (D) and the nitrogen imine group as acceptor (A). The H–A distances are in the 1.77–1.81 Å range. **AzoNH**, **MeAzoNH**, and **ClAzoNH** showed weak interactions involving the methyl group and the π‐system of the benzylidenimine (Figure 6). The distances for these interactions are between 2.85 and 2.90(2) Å. Two π–π weak interactions can be observed between the pyrazol and the benzylidenimine π‐systems, with distances of 4.42 – 4.47 Å and 4.89 – 4.92 Å. In contrast, in **MeOAzoNH**, the distances for these π‐π weak interactions (4.038(2) Å) are shorter. Crystal packing of **MeAzoNH**, **MeOAzoNH**, and **ClAzoNH** are shown in Figure S21.

### Frontier Molecular Orbitals (FMO)

The most critical orbitals in a molecule are the frontier molecular orbitals, corresponding to the highest occupied molecular orbital (HOMO) and lowest unoccupied molecular orbital (LUMO). The frontier orbital gap helps characterizing the molecule‘s kinetic stability, chemical reactivity, and selectivity in terms of global parameters, such as electronegativity, hardness, and softness.^[36]^

Thus, each ground‐state structure was doped with an additional electron by setting the charge to −1 (the charge was set to +1 when calculating the ionization potential, IP). Single‐point calculations were used to obtain electron affinity (EA). IP and EA were calculated from equations 1a and 1b, where E^0^ is the ground‐state energy of the molecule, E^−1^ (HOMO^−1^) is the energy of the negatively charged ion, and

**Figure 7:**
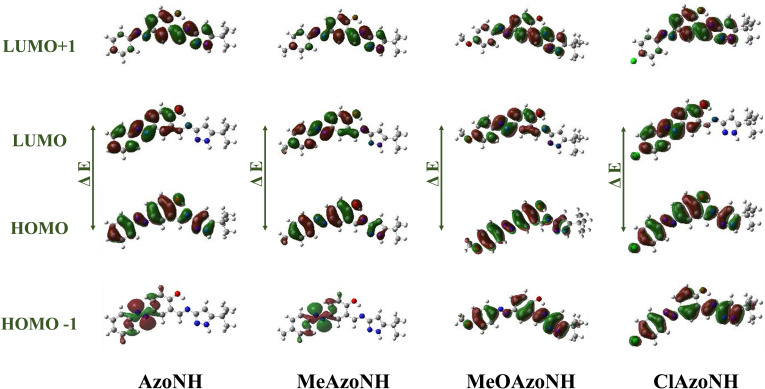
The frontier molecular orbitals (HOMOs and LUMOs) of **AzoNH**, **MeAzoNH**, **ClAzoNH**, and **MeOAzoNH**.

E^+1^ (LUMO^+1^) is the energy of the positively charged ion in the ground‐state geometry. Therefore, the band gap is conveniently obtained as the difference between IP and EA.^[37]^ The boundary orbitals HOMO, HOMO^−1^, LUMO, and LUMO^+1^ of the compounds were calculated using B3LYP/6‐311^++^G** (Figure 7).

The chemical hardness can be calculated as shown in equation 1d. Global softness is the inverse of hardness (equation 1e). Parr *et al*. introduced the global electrophilicity index (ω), which measures the propensity of a species to accept electrons, which can be calculated using the electronic system chemical potential (μ) as shown in equation 1c.^[38]^

(1a)

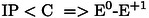



(1b)

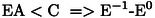

(1c) $$-\mu \;\approx c\;=\frac{IP+EA}{2}$$

(1d) $$\eta =\frac{IP-EA}{2}$$

(1e) $$\sigma =\frac{1}{h}$$

(1 f) $$\omega =\frac{\;m^{2}}{2\;\;h}$$

Comparing the values of hardness and softness allows for determining the relative reactivity. The HOMO‐LUMO energy gap, electronegativity, electrophilicity index, the chemical hardness and softness values of the compounds are listed in Table 1. According to B3LYP/6‐311^++^G** calculations, the energy band gap ΔE (transition from HOMO to LUMO) of the azomethine‐pyrazoles is about 3.43 to 3.56 eV. Data in Table 1 show that the **MeOAzoNH** compound is the most reactive as it presents the lowest energy difference value and, as such, could be more sensitive to reactions associated with electron transfer and interactions with biological systems. **MeAzoNH** displays the highest value of ΔE and the highest value of hardness, which makes it less reactive. In contrast, the high value of the electrophilicity index for **ClAzoNH** makes it a better electrophile than the other compounds.

**Table 1:** HOMO‐LUMO energies and calculated global chemical parameters of compounds **AzoNH**, **MeAzoNH**, **MeOAzoNH**, and **ClAzoNH**.

Parameters	AzoNH	MeAzoNH	MeOAzoNH	ClAzoNH
E_LUMO_^+1^	−2.20	−1.95	−2.14	−2.21
E_LUMO_	−2.67	−2.27	−2.54	−2.74
E_HOMO_	−6.19	−5.83	−5.97	−6.19
E_HOMO_^−1^	−6.70	−6.26	−6.59	−6.75
ΔE	3.52	3.56	3.43	3.45
c	4.43	4.05	4.25	4.46
η	1.76	1.78	1.71	1.72
σ	0.57	0.56	0.58	0.58
ω	5.57	4.61	5.28	5.78

* c: chemical potential, η : chemical hardness, σ : global softness and ω: electrophilicity index.

### In Vitro Studies on the Antifungal Activity

All new compounds were tested against clinically significant fungal species *C. albicans* and *C. neoformans*. Each compound was dissolved in RPMI‐1640 at concentrations between 250 and 1.7 μg/mL. Amphotericin B was used as a positive control, for which 100 % inhibition was achieved for both strains in the entire range of concentrations.^[39]^ The corresponding Minimum Inhibitory Concentration (MIC) values were calculated from the graphs of the percentage of inhibition against the concentration for each compound in the different strains, as shown in Figures S18‐S21. **AzoNH**, **MeAzoNH**, **MeOAzoNH**, and **ClAzoNH** exhibited MIC_**50**_ values of 5.91, 6.25, >125, and 2.08 μg/mL for *C. albicans*, respectively, and 2.83, 2.08, 9.16, and 23.08 μg/mL for *C. neoformans*, respectively (Table 2). Although **ClAzoNH** was the most active compound against *C. albicans*, it was also the least active for *C. neoformans*. On the other hand, the compound **AzoNH** stands out as the second compound with the most significant activity against both strains.

**Table 2:** MIC values for azomethine‐pyrazole compounds on *C. albicans* (*C.a*) and *C. neoformans* (*C.n*).

Compound	Fungus	Concentration μg/mL		
		MIC_100_	MIC_80_	MIC_50_
**AzoNH**	*C.a*	30.92	7.58	5.91
	*C.n*	61.42	7.42	2.83
**MeAzoNH**	*C.a*	62.08	11.66	6.25
	*C.n*	30.83	7.50	2.08
**MeOAzoNH**	*C.a*	>125	>125	>125
	*C.n*	>125	48.08	9.16
**ClAzoNH**	*C.a*	11.66	2.50	2.08
	*C.n*	>125	86.25	23.08
**Amphotericin B**	*C.a*	1.00	0.50	0.25
	*C.n*	1.25	0.50	0.25

The excellent activity of the compounds **AzoNH**, **MeAzoNH**, **MeOAzoNH**, and **ClAzoNH** is based on the fact that they were designed taking into account our previous studies on nitrophenylpyrazole‐derived 2‐hydroxyphenyl Schiff bases, which displayed moderate activity against *C. albicans*.^[35]^ In such studies, the antifungal activity was dependent on the nitro group‘s position in the aromatic ring. This led to the synthesis of azomethine derivatives derived from nitrophenylpyrazoles,^[29]^ which exhibited enhanced antifungal activity compared to the Schiff bases derived from 2‐hydroxyphenyl nitrophenylpyrazoles. The pyrazole‐based azomethine compound (Azo 1, Figure 3)^[29]^ presented a 47 % inhibition on the *C. neoformans* strain with a concentration of 31.2 μg/mL, while its analog without the phenyl ring on the pyrazole moiety, AzoNH, showed a 50 % inhibition with a concentration of 2.83 μg/mL on the same strain (Table 2). The new compounds showed a superior antifungal activity towards *C. albinacans* compared to other pyrazoles, including pyrazole derived from indoles, pyrimidines, and phenylthioureas,^[40]^ benzothiazole pyrazole derivatives,^[41]^ pyrazoles derived from flavones and isoflavones,^[42]^ and pyridyl thiophenes and their Ni(II) complexes.^[43]^

Since the compounds **AzoNH**, **MeAzoNH**, and **ClAzoNH** showed high cytotoxic activity against *C. albicans*, their activity was evaluated on four different *Candida* strains, *Candida tropicalis* (*C.t*), *Candida glabrata* (*C.g*), *Candida parapsilosis* (*C.p*) and *Candida krusei* (*C.k*). Compound **AzoNH** was the most active against the 4 strains with MIC_50_ of 6.83 (*C.t*), 11.00 (*C.g*), 10.33 (*C.p*) and 5.75 (*C.k*) μg/mL (Table 3). While neither **MeAzoNH** nor **ClAzoNH** showed antifungal activity against *C. glabrata*, **AzoNH** showed high antifungal activity against *C. glabrata*. The results of our *in vitro* assays suggest that the **AzoNH** compound is a promising scaffold for future studies and new searches for organic compounds with antifungal activity.

**Table 3:** MIC values for **AzoNH**, **ClAzoNH**, and **MeAzoNH** compounds on different *Candida* strains. Amphotericin B was used as a positive control*.

Compound	Fungus	Concentration μg/mL		
		MIC_100_	MIC_80_	MIC_50_
**AzoNH**	*C.t*	>125	13.66	6.83
	*C.g*	>125	14.25	11.00
	*C.p*	62.5	45.33	10.33
	*C.k*	>125	7.91	5.75
**MeAzoNH**	*C.t*	>125	57.5	15.25
	*C.g*	>125	>125	>125
	*C.p*	>125	84.50	14.58
	*C.k*	>125	97.25	52.5
**ClAzoNH**	*C.t*	>125	108	28.58
	*C.g*	>125	>125	>125
	*C.p*	>125	51.25	25.42
	*C.k*	>125	>125	>125

*MIC_100_ for *C.t*=0.50 μg/mL, *C.g*=0.39 μg/mL, *C.p*=0.78 μg/mL, and *C.k*=0.39 μg/mL

### Molecular Docking Modeling

To obtain insights into the possible mechanism of action of the new azomethine‐pyrazoles, molecular docking calculations between our compounds and a series of proteins described as targets of azo compounds in fungi were performed. The results of the docking scores for each compound in each protein and the two fungi (*C. albicans and C. neoformans*) are shown in Tables 4 and 5.

**Table 4:** Molecular docking results for *C. albicans*. Compounds are highlighted in green, yellow, orange, and red according to the scores obtained by each software for binding affinity (kcal/mol); the more negative values mean a higher affinity.

**Compound**	**Software**	***Ca*** **CYP51**	***Ca*** **NMT**	***Ca*** **TMPK**
**AzoNH**	A. vina	−9.40±0.00	−10.30±0.00	−9.90±0.00
	Smina	−9.60±0.06	−10.58±0.04	−10.12±0.04
**MeAzoNH**	A. vina	−9.10±0.00	−9.90±0.00	−9.68±0.00
	Smina	−9.30±0.00	−10.18±0.04	−9.90±0.00
**MeOAzoNH**	A. vina	−9.02±0.40	−9.70±0.00	−9.30±0.10
	Smina	−9.20±0.00	−9.90±0.00	−9.56±0.04
**ClAzoNH**	A. vina	−9.22±0.04	−9.90±0.00	−9.66±0.04
	Smina	−9.48±0.09	−10.10±0.00	−9.99±0.00

**Table 5:** Molecular docking results for *C. neoformans*. Compounds are highlighted as described for Table 4.

**Compound**	**Software**	***Cn*** **FTase**	***Cn*** **Hsp90**	***Cn*** **AdSS**
**AzoNH**	A. vina	−8.12±0.90	−7.92±0.07	−8.70±0.00
	Smina	−8.03±0.00	−8.08±0.04	−8.90±0.00
**MeAzoNH**	A. vina	−8.36±0.10	−7.78±0.04	−9.00±0.00
	Smina	−8.38±0.13	−7.90±0.00	−9.20±0.00
**MeOAzoNH**	A. vina	−7.92±0.07	−7.76±0.04	−9.20±0.00
	Smina	−8.04±0.04	−7.60±0.00	−9.38±0.04
**ClAzoNH**	A. vina	−8.02±0.04	−7.76±0.04	−9.00±0.00
	Smina	−8.24±0.04	−7.94±0.04	−9.20±0.00

**Figure 8:**
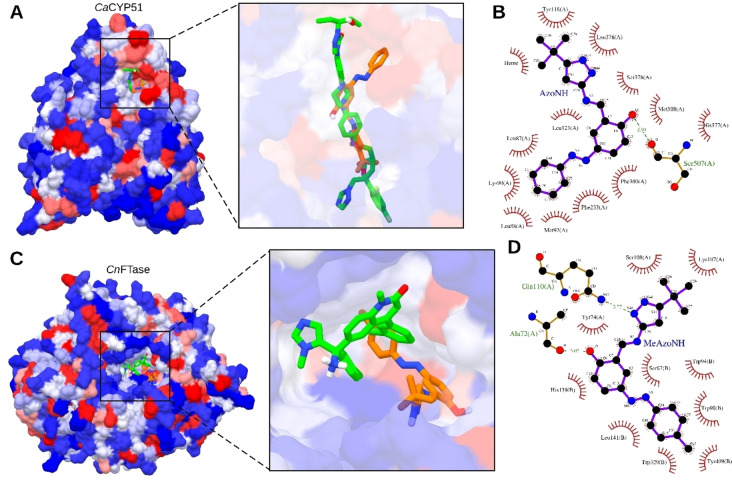
Molecular docking results between *Ca*CYP51‐**AzoNH** (**A**, **B**) and *CnFTase*‐**MeAzoNH** (**C**, **D**). (**A**)Visualization of posaconazole (green sticks) and **AzoNH** (orange sticks) poses inside *Ca*CYP51; the protein is shown in surface and colored according to its hydrophobicity scale, where blue color means hydrophilic amino acids and red color means hydrophobic amino acids. (**B)** 2D interactions between **AzoNH** and the active site of *Ca*CYP51. (**C**) Visualization of the crystallized inhibitor with an imidazole fragment (green sticks) and the **MeAzoNH** (orange sticks) poses inside the protein *Cn*FTase; the protein is represented as in **A**. (**D**) 2D interactions between **MeAzoNH** and the active site of *Cn*FTase. In **B** and **D**, the hydrophobic interactions are represented by red semi‐circles and dashed green lines represent H‐bonds. These results correspond to the best pose predicted by Autodock Vina.

For *C. albicans*, the protein that best represented the trend of the *in vitro* results (**ClAzoNH** and **AzoNH** were more active than **MeAzoNH** and **MeOAzoNH**) was the *Ca*CYP51 (sterol 14‐alpha demethylase) protein (Table 4), suggesting that CYP51 would be more likely to be a target in *C. albicans*. Compounds **ClAzoNH** and **AzoNH** presented the lowest MIC values towards *C. albicans* and the highest binding affinities for CYP51. It is worth mentioning that **MeOAzoNH** presented the lowest activity against *C. albicans* (Table 2) and the lowest binding affinity for CYP51 (Table 4). Such results are consistent with other experimental studies that have shown that CYP51 acts as a target for compounds derived from an azole nucleus.[^44^, ^45^, ^46^] All the compounds were docked inside the binding site of the posaconazole inhibitor (Figure 8A).^[29]^ The compound with the higher binding affinity for *Ca*CYP51 was **AzoNH** (Table 4), presenting interactions with residues such as Leu121, Phe233, His377, and Ser378, which have been described to interact with other inhibitors inside of CYP51.^[45]^ Additionally, **AzoNH** presented an *H*‐bond with the carbonyl group from the backbone of Ser507 (Figure 8B). Since the main structural difference between **AzoNH** and **MeOAzoNH** is the presence in the *para* position of a methoxy substituent, we deduced that interactions between **AzoNH** and hydrophobic residues such as Leu87, Leu88, Met92, and Phe233 could play a role in stabilizing its anchoring in the protein. The *tert*‐butyl group from **AzoNH** interacts with the Heme group from *Ca*CYP51, meaning this substituent could be of interest in future studies to enhance its affinity with the receptor.

Therefore, molecular docking calculations support the *in vitro* results that the compound **AzoNH** could be considered a promising starting point for further rational design of molecules with antifungal properties.

On the other hand, for *C. neoformans*, the protein that best reflects the *in vitro* assays is the *Cn*FTase (farnesyltransferase) protein, showing a greater affinity with **MeAzoNH** and **AzoNH** (Table 5). Figures 8C and D display the interaction modes with **MeAzoNH**, which showed the highest biological activity against *C. neoformans* (Table 2) and binding affinity values for the active site of the *Cn*FTase protein (Table 5). Noteworthy, the tolyl ring of **MeAzoNH** interacts with hydrophobic residues such as Trp90, Trp94, Leu141, Trp329, and Tyr409 (all of them present in the B chain or subunit 2 of the FTase protein). Additionally, **MeAzoNH** presented two hydrogen bonds with Ala72 and Gln110 residues of the A chain or subunit of the FTase protein (Figure 8D).

## Conclusions

The *para* substitution of the azo‐phenyl moiety (−N=N−) by chloride, methoxy, and methyl groups in a series of azomethyl‐pyrazole derivatives allowed the control of properties such as the energy difference between the HOMO and LUMO orbitals. The **MeAzoNH** compound displayed the highest ΔE and hardness values, while **ClAzoNH** was more electrophilic than the other pyrazoles. The *in vitro* antifungal assays showed that compounds **AzoNH** and **ClAzoNH** presented low MIC values against the prevalent and severe *C. albicans* strain. Additionally, **AzoNH** also displayed notable activity against four different *Candida* strains. In contrast, **MeOAzoNH** was the least active against *Candida* strains and *C. neoformans*. Molecular docking calculations confirmed the *in vitro* findings and allowed the identification of potential biological targets of the new azomethine‐pyrazoles. In the case of *C. albicans*, the *Ca*CYP51 protein seems to be a plausible target, while for *C. neoformans*, interactions with the *Cn*FTase protein better explained the trends observed *in vitro*. However, as molecular docking calculations serve as an initial approximation to predict the potential targets, more precise calculations are needed to strengthen our findings. Our interdisciplinary work highlights the importance of the rational design of bioactive compounds based on the azomethine‐pyrazole scaffold as antifungal agents, in which the compound **AzoNH** emerges as the most compelling candidate for further studies.

## Experimental

### Analytical and Physicochemical Measurements

All chemicals and solvents (analytical grade) were purchased from Sigma Aldrich and Across and used without further purification. The 5‐(*tert*‐butyl)‐1*H*‐pyrazol‐3‐amine aminopyrazole and azo‐aldehydes precursors were prepared according to the literature.[^30^, ^45^] The reactions were monitored by thin layer chromatography (TLC) using silica gel 60 F_264_ (Merck) alumina plates. The melting points were determined using a Büchi melting point apparatus. The excitation and emission spectra were obtained in a JASCO 8600 fluorescence spectrophotometer with an FMP‐825 microplate reader. Infrared spectra were recorded on an Alpha ATR spectrometer from Bruker. The NMR spectra were recorded on a Bruker Avance 400 spectrophotometer operating at 400 MHz for ^1^H and at 100 MHz for ^13^C and a Bruker Avance 300 spectrophotometer operating at 300 MHz for ^1^H and at 75 MHz for ^13^C, using tetramethylsilane as the internal standard. NMR spectra splitting patterns were as s (singlet), d (doublet), t (triplet), or bs (broad singlet). All chemical shifts (δ) are quoted as parts per million, and the coupling constants (*J*) are in Hertz (Hz). The mass spectra were obtained on a Shimadzu‐GCMS 2010‐DI‐2010 spectrometer equipped with a direct input probe operating at 70 eV. The UV–visible absorption spectra were obtained in a 200–600 nm range using a Shimadzu 160 spectrophotometer.

### General Method for the Synthesis of Schiff Bases Derivatives

Compounds 2‐((*E*)‐((5‐(*tert*‐butyl)‐1*H*‐pyrazol‐3‐yl)imino)methyl)‐4‐((*E*)‐phenyldiazenyl)phenol (**AzoNH**), 2‐((*E*)‐((5‐(*tert*‐butyl)‐1*H*‐pyrazol‐3‐yl)imino)methyl)‐4‐((*E*)‐*p*‐tolyldiazenyl)phenol (**MeAzoNH**), 2‐((*E*)‐((5‐(*tert*‐butyl)‐1*H*‐pyrazol‐3‐yl)imino)methyl)‐4‐((*E*)‐(4‐methoxyphenyl)diazenyl)phenol (**MeOAzoNH**) and 2‐((*E*)‐((5‐(*tert*‐butyl)‐1*H*‐pyrazol‐3‐yl)imino)methyl)‐4‐((*E*)‐(4‐chlorophenyl)diazenyl)phenol (**ClAzoNH**) were synthesized as follows.

A mixture of 5‐(*tert*‐butyl)‐1*H*‐pyrazol‐3‐amine aminopyrazole (1.44 mmol), the corresponding azo‐aldehyde (1.44 mmol), and glacial acetic acid (5 drops) was triturated with a spatula for 10 min at room temperature. After complete reaction (monitored by TLC), the resulting solid was washed with cold water (5×20 mL), filtered, and dried under vacuum to give the pure compounds.

### 2‐((E)‐((5‐(tert‐butyl)‐1H‐pyrazol‐3‐yl)imino)methyl)‐4‐((E)phenyldiazenyl)phenol (AzoNH)

**Figure 9:**
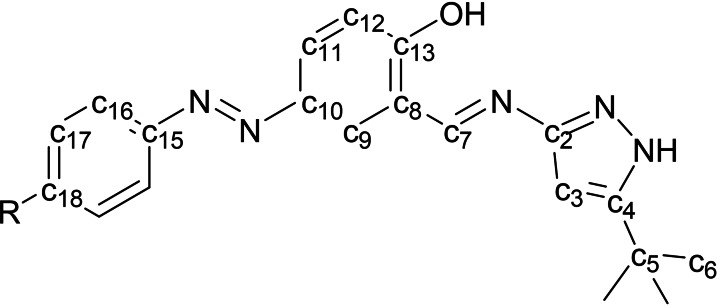
Numbering scheme used for NMR assignment. R= H (**AzoNH**), Me (**MeAzoNH**), MeO (**MeOAzoNH**), Cl (**ClAzoNH**).

Yellow solid. Yield 95 %. M.p. 210 – 212 °C. MS (70 eV) *m/z* (%) 347 [*M* ^*+*^] (100), 242 [*M* ^*+*^‐105] (86.88), 77 [*M* ^*+*^‐270] (29.55) 57 [*M* ^*+*^‐290] (13.54). UV‐Vis, MeCN, λ max nm: λ_1_ 245, λ_2_ 270, λ_3_ 327, λ_4_ 350. FT‐IR ATR (cm^−1^) ν(N−H) 3231, ν(C−H) 3131 (pyrazole), ν(C−H) 3064 (aromatic), ν_as_(C−H) 2954 (*t*‐butyl), ν_s_(C−H) 2866 (*t*‐butyl), ν(C=N) 1604 (imine), ν_as_(C=C) 1566 (aromatic), ν_s_(C=C) 1281 (aromatic), ν(N=N) 1473 (azo), ν(C−O) 1260. ^1^H‐NMR (300 MHz, acetone‐*d_6_*) 1.37 (s, 9H, *t*Bu−H), 6.38 (s, 1H, H‐3), 7.10 (d. 1H, ^*3*^ *J*=8.80, H‐12), 7.53 (t, 1H, ^*3*^ *J*=7.04, H‐18) 7.55 (t, 2H, ^*3*^ *J*=7.04, H‐17), 7.88 (d, 2H, ^*3*^ *J*=7.04, H‐16) 8.01 (dd, 2H, ^*3*^ *J*=8.8, ^*4*^ *J* =2.3, H‐11) 8.18 (d, 1H, ^*4*^ *J*=2.1, H‐9), 9.21 (s, 1H, H‐7). ^13^C‐NMR (75 MHz, acetone‐*d_6_*,) 29.42 (*t*‐Bu−C), 31.77 (C‐5), 93.03 (C‐3), 118.51 (C‐12), 120.10 (C‐8), 123.18 (C‐16), 123.51 (C‐11), 127.28 (C‐10), 129.02 (C‐9), 129.97 (C‐17), 131.44 (C‐18), 146.28 (C‐15), 153.33 (C‐4), 155.91 (C‐2), 162.43 (C‐7), 164.4 (C‐13). The atoms were numbered according to Figure 9. Elemental analysis for C_20_H_21_N_5_O calcd C, 69.14; H, 6.09; N, 20.16. Found: C, 69.16; H 6.07; N, 20.13.

### 2‐((E)‐((5‐(tert‐butyl)‐1H‐pyrazol‐3‐yl)imino)methyl)‐4‐((E)‐p–tolyldiazenyl)phenol (MeAzoNH)

Yellow solid. Yield 90 %. M.p. 204 – 206 °C. MS (70 eV) *m/z* (%) 361 [*M* ^*+*^] (100), 344 [*M* ^*+*^‐17] (18.39), 242 [*M* ^*+*^‐119] (80.47) 91 [*M* ^*+*^‐270] (57.84). UV‐Vis, MeCN, λ max nm: λ_1_ 231, λ_2_ 330, λ_3_ 352. FT‐IR ATR (cm^−1^) ν(N−H) 3228, ν(C−H) 3131 (pyrazole), ν_as_(C−H) 2966 (*t*‐butyl), ν_s_(C−H) 2867 (*t*‐butyl), ν(C=N) 1600 (imine), ν(C−O) 1240. ^1^H‐NMR (400 MHz, DMSO‐*d_6_*) 1.33 (s, 9H, *t*Bu−H), 2.42 (s, 3H, Me−H), 6.39 (s, 1H, H‐3), 7.15 (d. 1H, ^*3*^ *J*=8.80, H‐12), 7.40 (d, 2H, ^*3*^ *J*=7.90, H‐17), 7.79 (d, 2H, ^*3*^ *J*=7.90, H‐16), 7.98 (d, 1H, ^*3*^ *J*=8.80, H‐11), 8.24 (s, 1H, H‐9), 9.24 (s, 1H, H‐7), 12.68 (s, 1H, OH), 13.86 (bs, 1H, NH). ^13^C‐NMR (100 MHz, DMSO‐*d_6_*) 21.40 (Me−C), 30.30 (*t*‐Bu−C), 31.3 (C‐5), 92.1 (C‐3), 118.51 (C‐12), 120.10 (C‐2), 123.18 (C‐16), 123.51 (C‐13), 127.28 (C‐11), 129.02 (C‐9), 129.97 (C‐17), 131.44 (C‐18), 146.28 (C‐8), 153.33 (C‐15), 155.91 (C‐4), 162.43 (C‐7), 164.4 (C‐10). The atoms were numbered according to Figure 9. Elemental analysis for C_21_H_23_N_5_O calcd C, 69.78; H, 6.41; N, 19.38. Found: C, 69.66; H 6.59; N, 19.48.

### 2‐((E)‐((5‐(tert‐butyl)‐1H‐pyrazol‐3‐yl)imino)methyl)‐4‐((E)‐(4 methoxyphenyl)diazenyl)phenol (MeOAzoNH)

Brown solid. Yield 89 %. M.p. 218 – 220 °C. MS (70 eV) *m/z* (%) 377 [*M* ^*+*^] (100), 360 [*M* ^*+*^‐17] (24.39), 242 [*M* ^*+*^‐135] (59.47) 107 [*M* ^*+*^‐270] (70.16). UV‐Vis, MeCN, λ max nm: λ_1_ 249, λ_2_ 359. FT‐IR ATR (cm^−1^) ν(N−H) 3228, ν(C−H) 3131 (pyrazole), ν_as_(C−H) 2961 (*t*‐butyl), ν_s_(C−H) 2840 (*t*‐butyl), ν(C=N) 1600 (imine), ν(C−O) 1240. ^1^H‐NMR (400 MHz, CDCl_3_) 1.38 (s, 9H, *t*Bu−H), 3.89 (s, 3H, MeO−H), 6.19 (s, 1H, H‐3), 7.02 (d, 2H, ^*3*^ *J*=9.01, H‐17), 7.12 (d, 1H, H‐12), 7.87 (d, 2H, ^*3*^ *J*=9.02, H‐16), 7.99 (m, 2H, H‐11, H‐9), 9.00 (s, 1H, H‐7), 13.49 (bs, 1H, NH). ^13^C‐NMR (100 MHz, CDCl_3_) 30.10 (*t*‐Bu−C), 31.34 (C‐5); 55.59 (MeO−C), ^13^C NMR (75 MHz, CDCl_3_) δ 30.10 (*t*‐Bu−C), 31.34 (C‐5), 55.59 (MeO−C), 93.43 (C‐3), 114.24 (C‐17), 118.04 (C‐12), 118.85 (C‐8), 124.43 (C‐16), 127.07 (C‐11), 127.67 (C‐9), 145.70(C‐15), 146.93 (C‐10), 155.57 (C‐4), 155.93(C‐2), 161.72 (C‐18), 162.19 (C‐7), 163.41(C‐13). The atoms were numbered according to Figure 9. Elemental analysis for C_21_H_23_N_5_O_2_ calcd C, 66.83; H, 6.14; N, 18.55. Found: C, 67.01; H 5.96; N, 18.33.

### 2‐((E)‐((5‐(tert‐butyl)‐1H‐pyrazol‐3‐yl)imino)methyl)‐4‐((E)‐(4‐chlorophenyl)diazenyl)phenol (ClAzoNH)

Brown solid. Yield 96 %. M.p. 205 – 207 °C. MS (70 eV) *m/z* (%) 381 [*M* ^*+*^] (92.65), 364 [*M* ^*+*^‐17] (22.39), 242 [*M* ^*+*^‐139] (100) 111 [*M* ^*+*^‐270] (33.91). FT‐IR ATR (cm^−1^): ν(N−H) 3248, ν(C−H) 3131 (pyrazole), ν_as_(C−H) 2961 (*t*‐butyl), ν_s_(C−H) 2866 (*t*‐butyl), ν(C=N) 1614 (imine), ν(C−O) 1249. UV‐Vis MeCN, λ max nm: λ_1_ 249, λ_2_ 328, λ_3_ 355. ^1^H‐NMR (400 MHz, CDCl_3_) 1.320 (s, 9H, *t*Bu−H), 6.126 (s, 1H, H‐3), 7.020 (m, 1H, ^*3*^ *J*=8.876, H‐12), 7.394 (d, 2H, ^*3*^ *J*=8.677, H‐16), 7.418 (d, 1H, H‐11), 7.749 (d, 2H, ^*3*^ *J*=8.652 H‐17), 7.790 (s, 1H, H‐9), 8.921 (s, 1H, H‐7), 9.952 (s, 1H, OH), 13.499 (bs, 1H, NH). ^13^C‐NMR (100 MHz, CDCl_3_) 30.093 (*t*‐Bu−C), 31.359 (C‐5), 93.552 (C‐3), 118.230 (C‐12), 118.855 (C‐8), 123.879 (C‐16), 127.270 (C‐11), 129.324 (C‐17), 129.433 (C‐9), 130.634 (C‐10), 136.371 (C‐18), 145.445 (C‐15), 150.956 (C‐4), 155.663 (C‐2), 162.140 (C‐13), 164.188 (C‐7). The atoms were numbered according to Figure 9. Elemental analysis for C_20_H_20_N_5_OCl calcd C, 62.91; H, 5.28; N, 18.34. Found: C, 63.11; H 5.17; N, 17.99.

### X‐Ray Diffraction Crystallography

Data for compounds were collected at room temperature (298 K) on a Bruker Apex‐II CCD diffractometer using monochromatic graphite MoKα (0.71073 Å) radiation. Cell determination and final cell parameters were obtained on all reflections using the Bruker SAINT software included in the APEX2 software suite. The integration and scaling of the data were carried out using the Bruker SAINT software. Data integration, Lorentz‐polarization effects, and absorption corrections were performed with CrysAlisPro.^[47]^ The crystalline structures were solved by direct methods using the Olex2 program.^[48]^ All the hydrogen atoms were placed in calculated positions and refined with fixed individual displacement parameters [Uiso(H)=1.2Ueq or 1.5Ueq] according to the riding model. An exception was made for the hydrogen atom from the hydroxyl group, which was located farther away from the electronic density. Molecular representations were generated by Diamond^[49]^ and MERCURY 3.9.^[50]^ The CIF files have been deposited in the Cambridge Structural Database under the codes CCDC 2827930 for **AzoNH**, 2327928 for **MeAzoNH**, 2327931 for **MeOAzoNH** and 2327929 for **ClAzoNH**. Copies of the data can be obtained, free of charge, at www.ccdc.cam.ac.uk.

### DFT Calculations

Theoretical calculations were determined using the Density Functional Theory (DFT) computational method, incorporating the Becke three‐parameter exchange couple with B3LYP with the base set 6–311^++^G^✶✶^ and Gaussian packet 09 without any obstacle to geometry.[^51^, ^52^] The calculated frequencies were corrected with the scale factor 0.960461 and then interpreted by means of potential energy distributions (PEDs) using the VEDA 4 program and the percentage contribution of the frontier orbitals at each transition of electronic absorption spectrum using GaussSum software.[^53^, ^54^, ^55^]

### Molecular Docking Calculations

Autodock Vina^[56]^ and Smina^[57]^ softwares were used for molecular docking calculations. Five replicates were performed for each molecular docking calculation, and the averages and standard deviations of the binding affinities are shown in Tables 4 and 5. The boxes in which the molecular dockings were performed had a dimension of 26×26×26 Å. All boxes were centered on the binding site of each control ligand (crystallized ligand), which validation for each of the proteins used as molecular targets was previously reported.^[29]^ All calculations were performed with a value for the exhaustiveness parameter equal to 15.

### Antifungal Assays

The compounds were tested against fungal species *C. albicans* (ATCC 10231) and *C. neoformans* (ATCC 32264), *C. tropicalis* (ATCC131), *C. glabrata* (ATCC 90030), *C. parapsilosi* (ATCC 7330) and *C. krusei* (ATCC 6258) from the American Type Culture Collection (ATCC), Rockville, MD, USA and CEREMIC (C), Centro de Referencia Micológica, Facultad de Ciencias Bioquímicas y Farmacéuticas, Suipacha 531‐(2000)‐Rosario, Argentina. The antifungal activity was determined using the standardized microbroth dilution method M‐27 A3 of the Clinical and Laboratory Standards Institute (CLSI).^[39]^ Compound test wells (CTWs) were prepared with stock solutions of each compound in DMSO (maximum concentration of DMSO≤1 %), diluted with RPMI‐1640, to final concentrations of 250–1.7 μg/mL. Amphotericin B (Sigma‐Aldrich) was used as a positive control. Tests were performed in triplicate. MIC_50_ corresponds to the concentration at which 50 % suppression of visible growth of the germinated *Candida* strains occurred, MIC_80_ is the concentration at which 80 % suppression of growth occurred, and MIC_100_, the lowest concentration of the compound at which fungal growth was suppressed entirely compared to the growth in the control well. Growth reduction for each compound concentration was calculated as follows: % of inhibition=100 ‐ (OD 405 CTW–OD 405 SCW)/(OD 405 GCW ‐ OD 405 SCW). The means ± SEM were used for constructing the dose‐response curves representing % inhibition vs concentration of each compound using Prism 9.5.0 software.

## Supplementary material

The IR, mass, and NMR spectra, graphs of percent inhibition *vs* concentration, and crystallographic data of the azomethine‐pyrazoles are available in supplementary data.

## Conflict of Interests

The authors declare no conflict of interest. The funders had no role in the design of the study; in the collection, analyses, or interpretation of data; in the writing of the manuscript, or in the decision to publish the results.

## Supporting information

As a service to our authors and readers, this journal provides supporting information supplied by the authors. Such materials are peer reviewed and may be re‐organized for online delivery, but are not copy‐edited or typeset. Technical support issues arising from supporting information (other than missing files) should be addressed to the authors.

Supporting Information

## Data Availability

The data supporting this article have been included as part of the Supplementary Information.
