# Supplementary material for: Evaluation of the Antifungal Properties of Azomethine‐Pyrazole Derivatives from a Structural Perspective
Source: ChemistryOpen. 2025 Apr 28;14(10):e202500132. doi: 10.1002/open.202500132 (PMC12518042; doi:10.1002/open.202500132)
Supplement: Supplementary file 1 — Supporting Information [file OPEN-14-e202500132-s001.pdf]

ChemistryOpen

Supporting Information

Evaluation of the Antifungal Properties of Azomethine-Pyrazole Derivatives from a Structural Perspective

María Isabel Murillo, Andrés Camilo Restrepo-Acevedo, Cristian Rocha-Roa, Susana Zacchino, Laura Svetaz, Simón Hernández-Ortega, Rodrigo Abonia, Ronan Le Lagadec,* and Fernando Cuenú-Cabezas*

Evaluation of the antifungal properties of azomethine-pyrazole derivatives from a structural perspective

María Isabel Murillo[a,b], Andrés Camilo Restrepo-Acevedo[a,b], Cristian Rocha-Roa[c,d], Susana Zacchino [e], Laura Svetaz[e], Simón Hernández-Ortega[b], Rodrigo Abonia[f], Ronan Le Lagadec[b]*, Fernando Cuenú-Cabezas[a]*

[a] Laboratorio de Química inorgánica y catálisis, Programa de Química, Universidad del Quindío, Carrera 15, Calle 12 Norte, Armenia, Colombia.

[b] Instituto de Química UNAM, Circuito Exterior s/n, Ciudad Universitaria, 04510 Ciudad de México, México.

[c] Grupo GEPAMOL, Centro de Investigaciones Biomédicas, Universidad del Quindío, Carrera 15, Calle 12 Norte, Armenia 630004, Colombia.

[d] Department of Biology, University of Fribourg, Fribourg CH-1700, Switzerland.

[e] Área Farmacognosia, Facultad de Ciencias Bioquímicas y Farmacéuticas, Universidad Nacional de Rosario, Suipacha 531, 2000 Rosario, Argentina.

[f] Departamento de Química, Universidad del Valle, Calle 13 No. 100-00, A.A. 25360, Cali, Colombia.

***Corresponding Authors:** Fernando Cuenú-Cabezas: fercuenu@uniquindio.edu.co; Ronan Le Lagadec: ronan@unam.mx

Contents

Figure S1. Mass Spectrometry of **AzoNH**.

Figure S2. IR spectrum of **AzoNH**.

Figure S3. ^{1}H-NMR spectrum of **AzoNH** in acetone-d_6.

Figure S4. ^{13}C-NMR spectrum of **AzoNH** in acetone-d_6.

Figure S5. Mass Spectrometry of **MeAzoNH**.

Figure S6. IR spectrum of **MeAzoNH**.

Figure S7. ^{1}H-NMR spectrum of **MeAzoNH** in DMSO-d_6.

Figure S8. ^{13}C-NMR spectrum of **MeAzoNH** in DMSO-d_6.

Figure S9. HSQC-NMR spectrum of **MeAzoNH** in DMSO-d_6.

Figure S10. HMBC-NMR spectrum of **MeAzoNH** in DMSO-d_6.

Figure S11. Mass Spectrometry of **MeOAzoNH**.

Figure S12. IR spectrum of **MeOAzoNH**.

Figure S13. ^{1}H-NMR spectrum of **MeOAzoNH** in $CDCl_3$.

Figure S14. ^{13}C-NMR spectrum of **MeOAzoNH** in $CDCl_3$.

Figure S15. HSQC-NMR spectrum of **MeOAzoNH** in $CDCl_3$.

Figure S16. Mass Spectrometry of **ClAzoNH**.

Figure S17. IR spectrum of **ClAzoNH**.

Figure S18. ^{1}H-NMR spectrum of **ClAzoNH** in $CDCl_3$.

Figure S19. ^{13}C-NMR spectrum of **ClAzoNH** in $CDCl_3$.

Figure S20. HSQC-NMR spectrum of **ClAzoNH** in $CDCl_3$.

Figure S21. Centroid-centroid interactions

Figure S22. Percentage inhibition *versus* concentration of **AzoNH** on *Candida tropicalis* (*C.t*), *Candida glabrata* (*C.g*), *Candida parapsilosi* (*C.p*), *Candida krusei* (*C.k*), *C. albicans* (*C.a*) and *C. neoformans* (*C.n*).

Figure S23. Percentage inhibition *versus* concentration of **ClAzoNH** on *Candida tropicalis* (*C.t*), *Candida glabrata* (*C.g*), *Candida parapsilosi* (*C.p*), *Candida krusei* (*C.k*), *C. albicans* (*C.a*) and *C. neoformans* (*C.n*).

Figure S24. Percentage inhibition *versus* concentration of **MeAzoNH** on *Candida tropicalis* (*C.t*), *Candida glabrata* (*C.g*), *Candida parapsilosi* (*C.p*), *Candida krusei* (*C.k*), *C. albicans* (*C.a*) and *C. neoformans* (*C.n*).

Figure S25. Percentage inhibition *versus* concentration of **MeOAzoNH** on *C. albicans* (*C.a*) and *C. neoformans* (*C.n*).

Table S1. Crystallographic data and refinement parameters

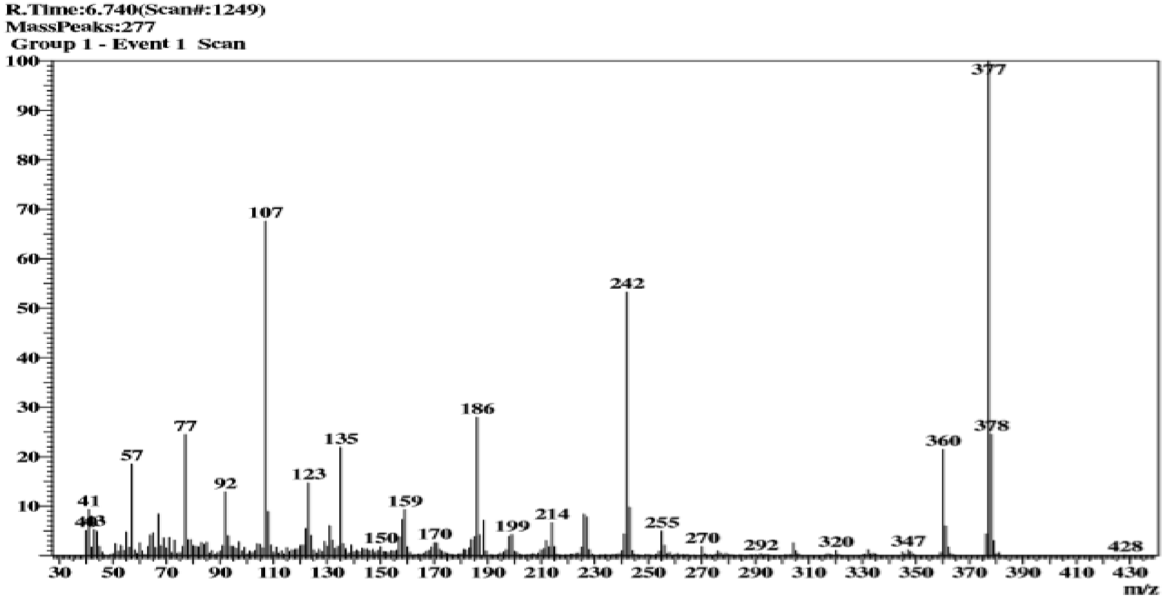

Figure S1. Mass Spectrometry of **AzoNH**.

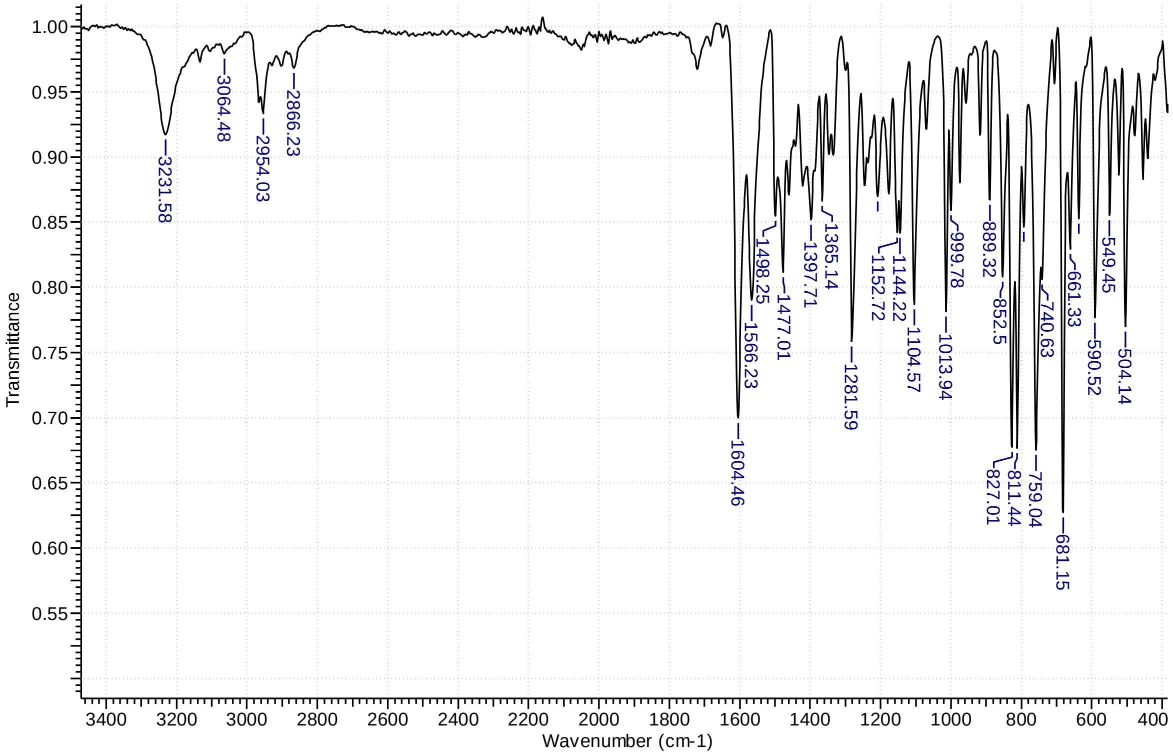

Figure S2. IR spectrum of **AzoNH**.

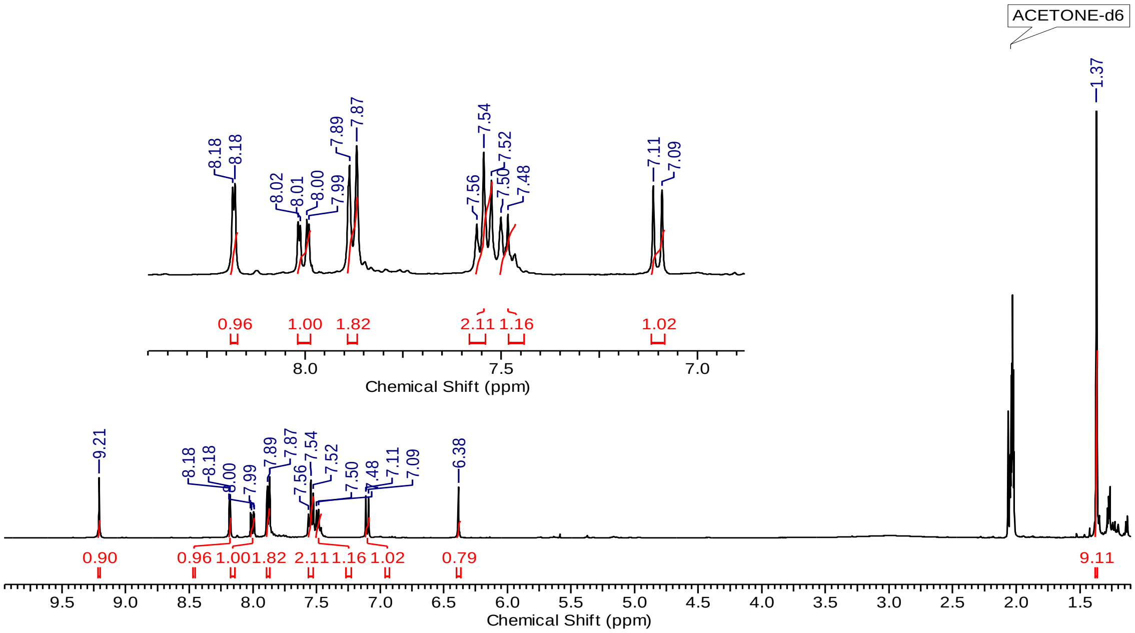

Figure S3. ^{1}H-NMR spectrum of **AzoNH** in acetone-d_6.

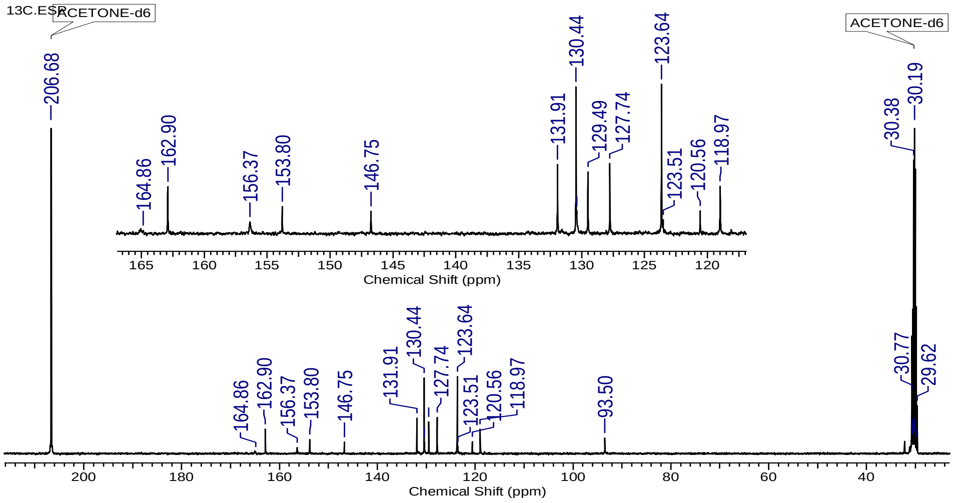

Figure S4. ^{13}C NMR spectrum of **AzoNH** in acetone-d_6.

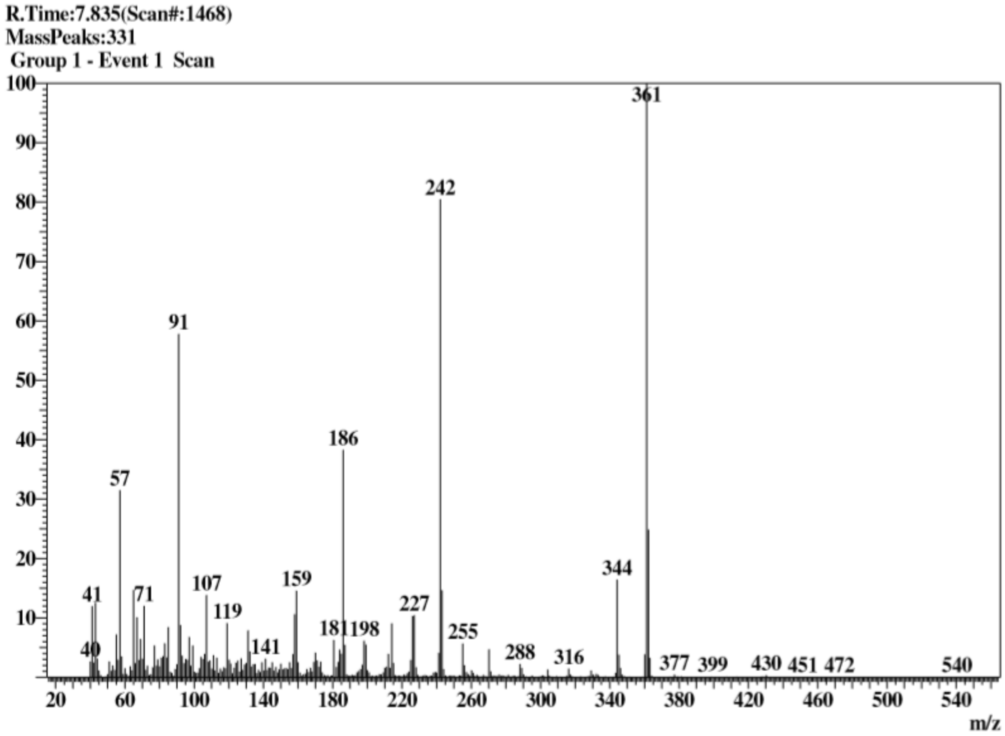

Figure S5. Mass Spectrometry of **MeAzoNH**.

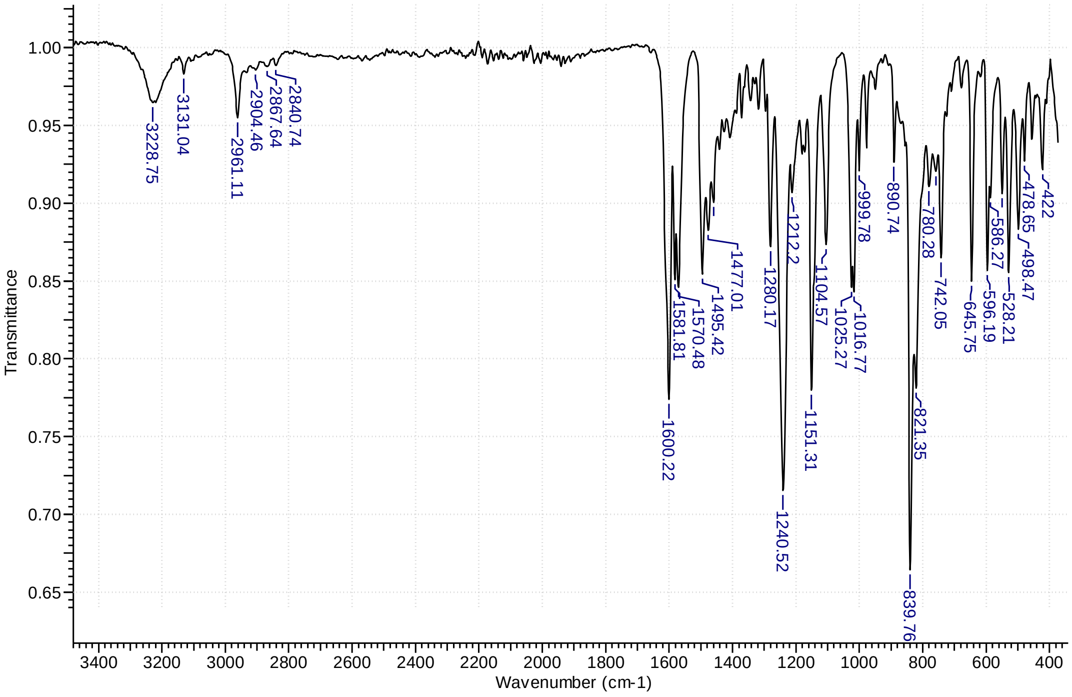

Figure S6. IR spectrum of **MeAzoNH**.

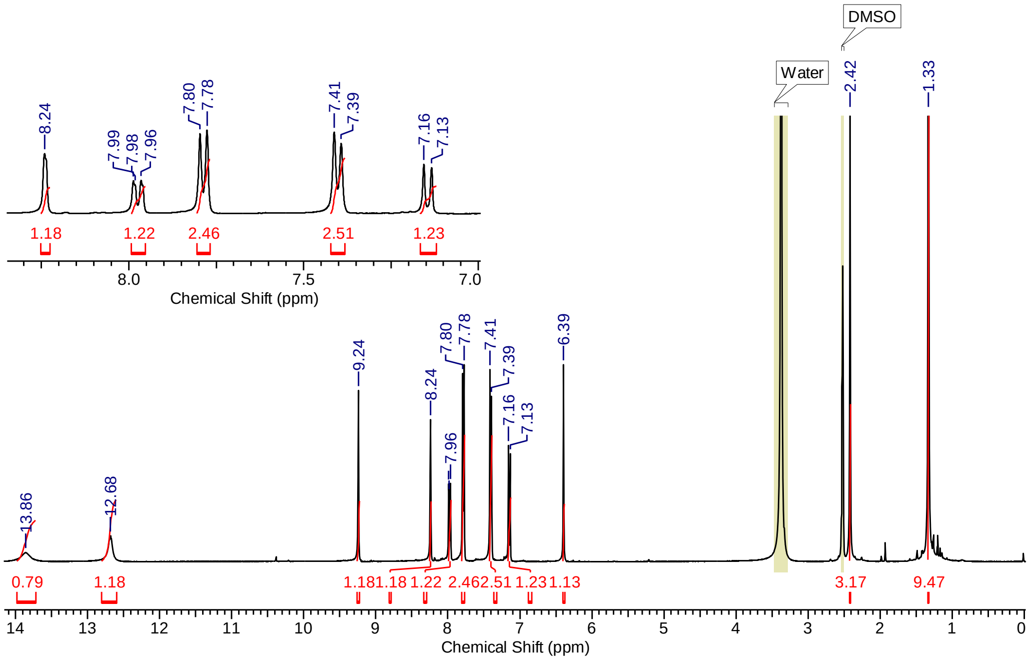

Figure S7. ^{1}H-NMR spectrum of **MeAzoNH** in DMSO-d_6.

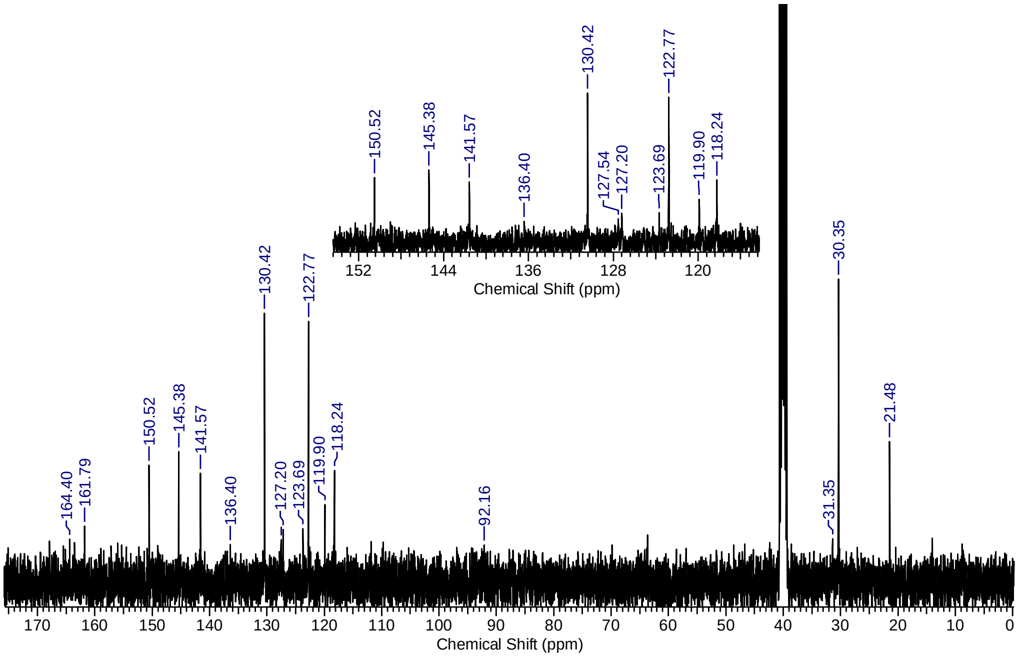

Figure S8. ^{13}C-NMR spectrum of **MeAzoNH** in DMSO-d_6.

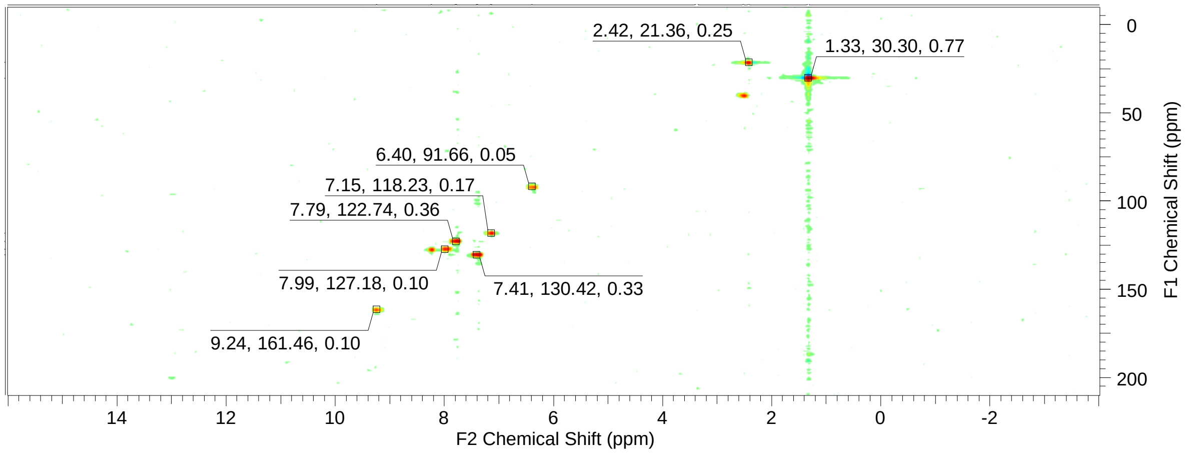

Figure S9. HSQC-NMR spectrum of **MeAzoNH** in DMSO-d_6.

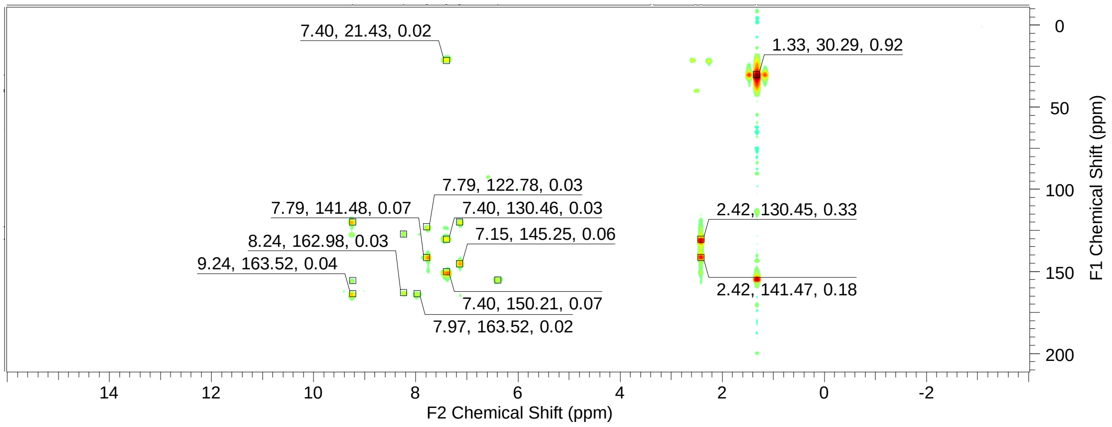

Figure S10. HMBC-NMR spectrum of **MeAzoNH** in DMSO-d_6.

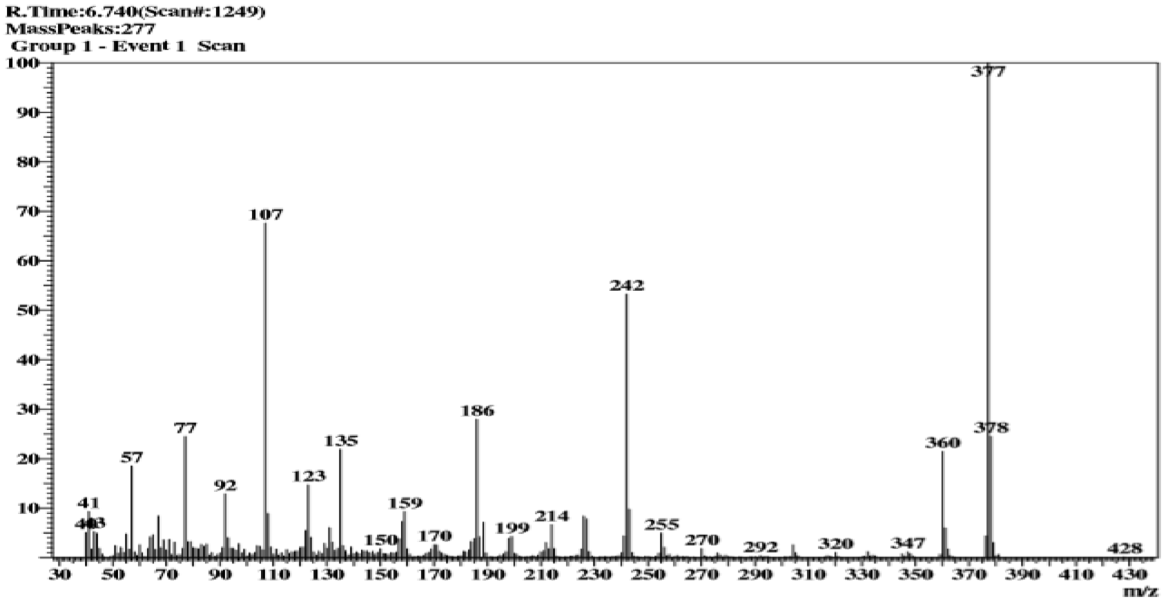

Figure S11. Mass Spectrometry of **MeOAzoNH**.

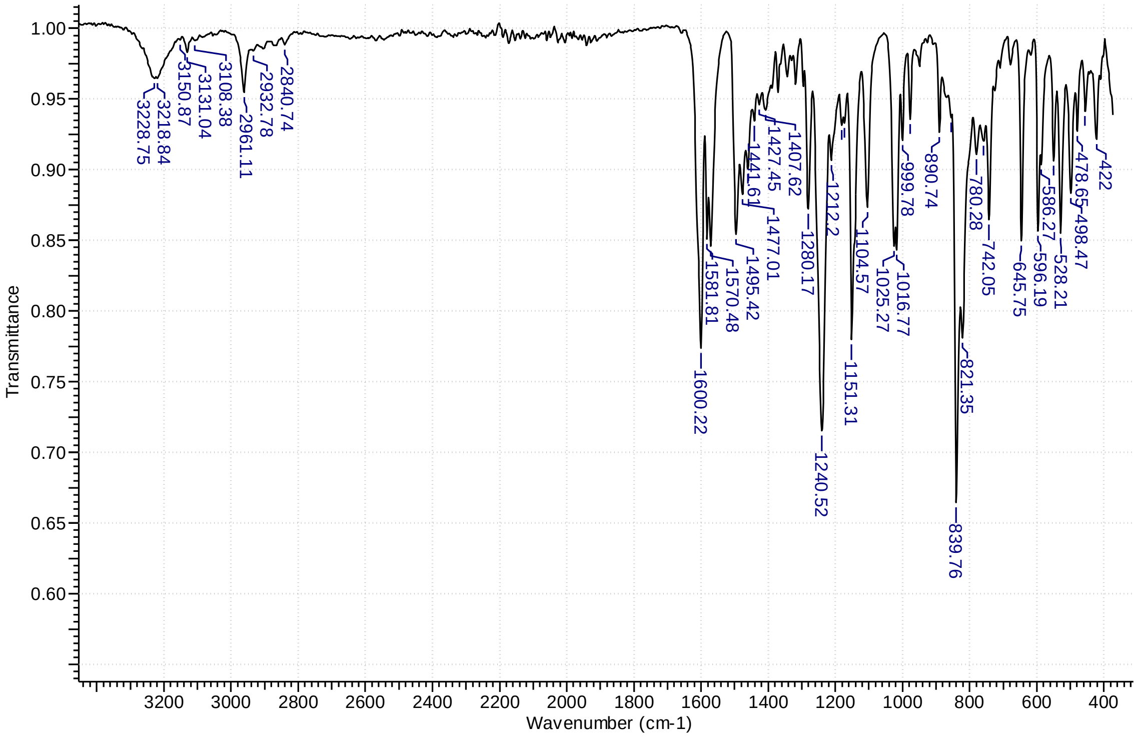

Figure S12. IR spectrum of **MeOAzoNH**.

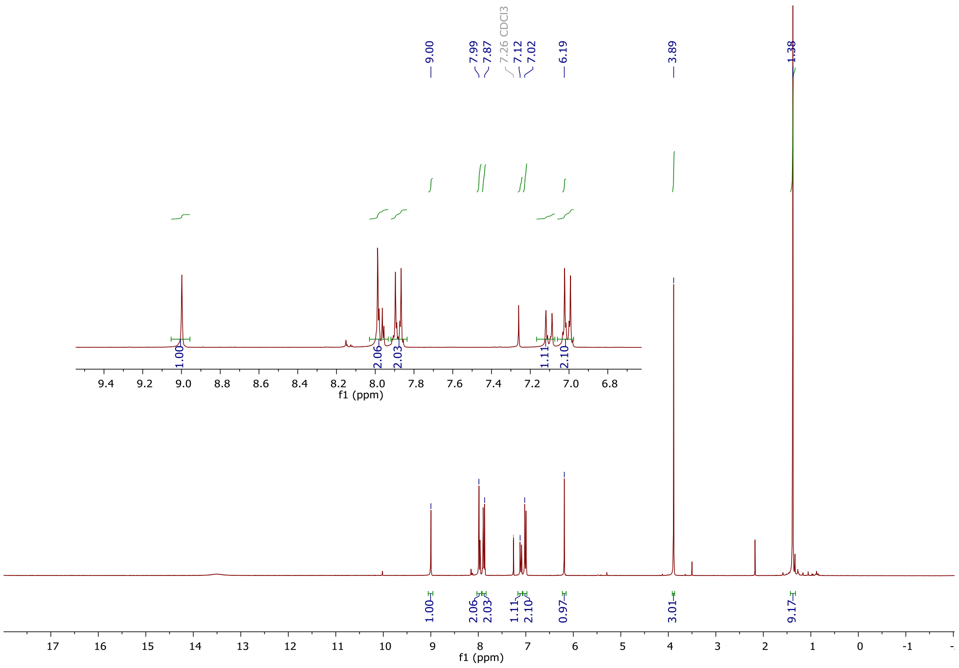

Figure S13. ^{1}H-NMR spectrum of **MeOAzoNH** in $CDCl_3$.

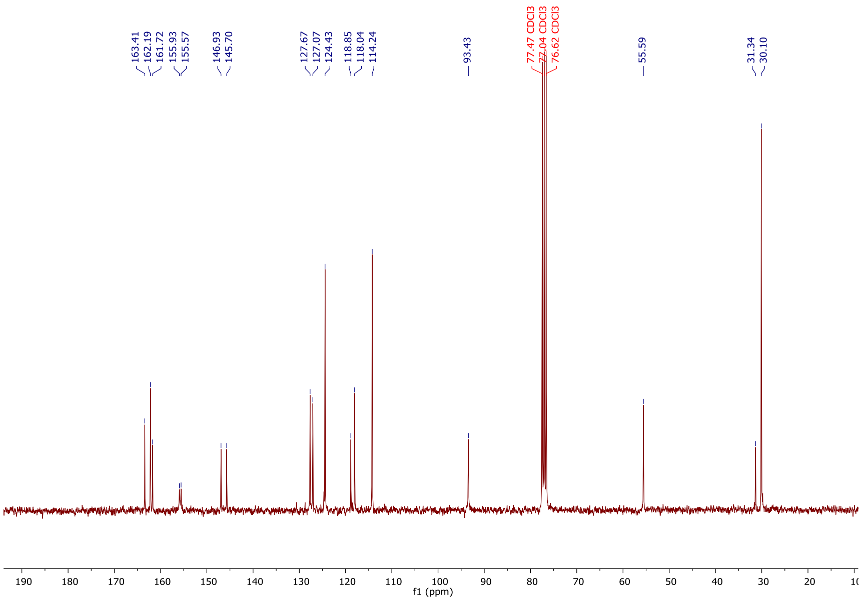

Figure S14. ^{13}C-NMR spectrum of **MeOAzoNH** in $CDCl_3$.

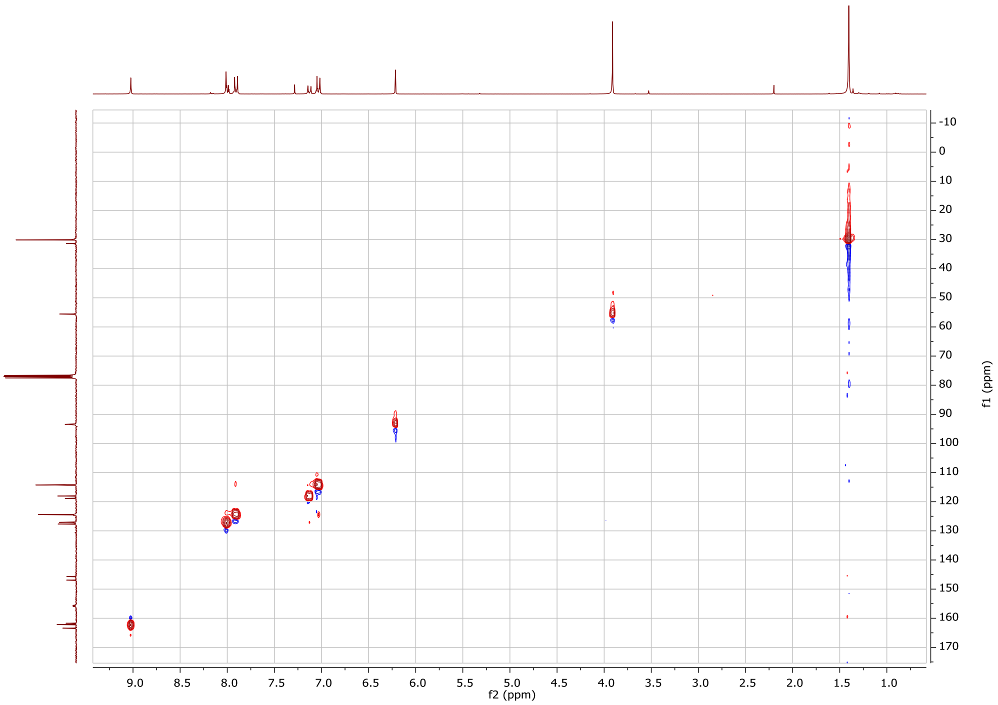

Figure S15. HSQC-NMR spectrum of **MeOAzoNH** in $CDCl_3$.

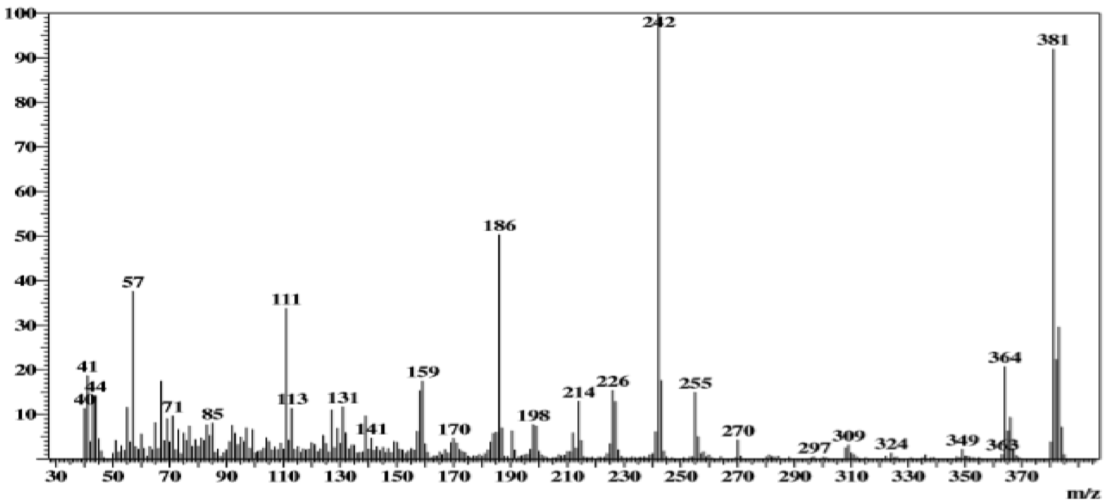

Figure S16. Mass Spectrometry of **ClAzoNH**.

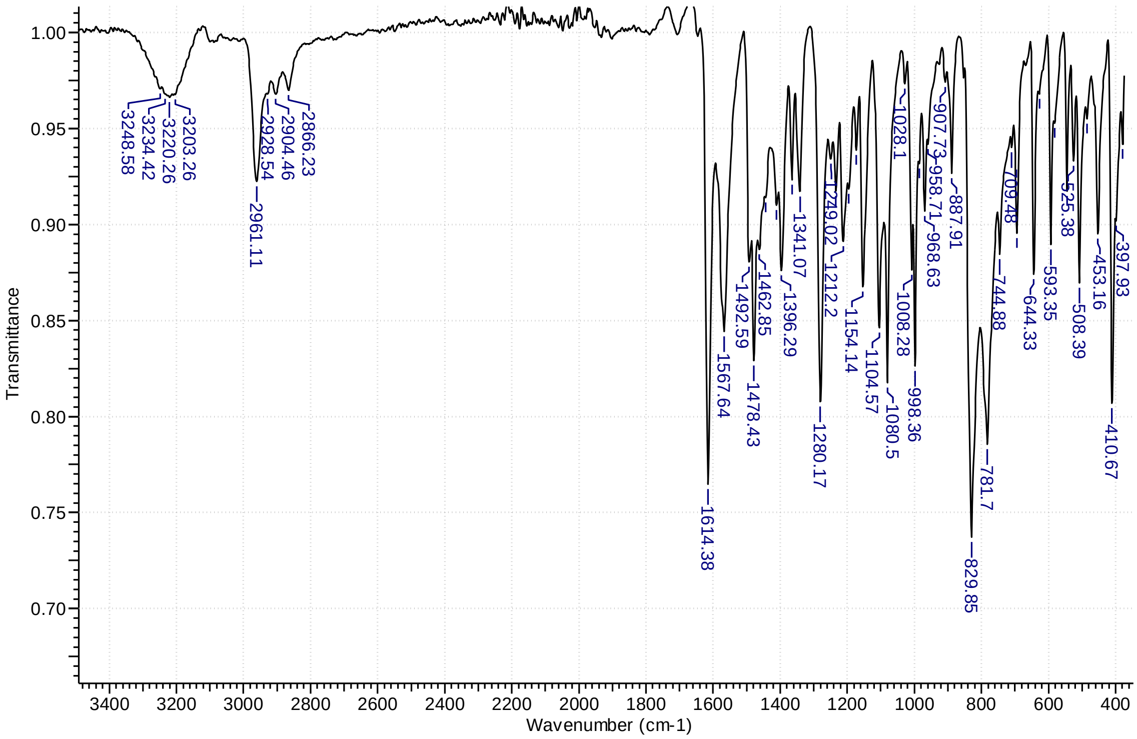

Figure S17. IR spectrum of **ClAzoNH**.

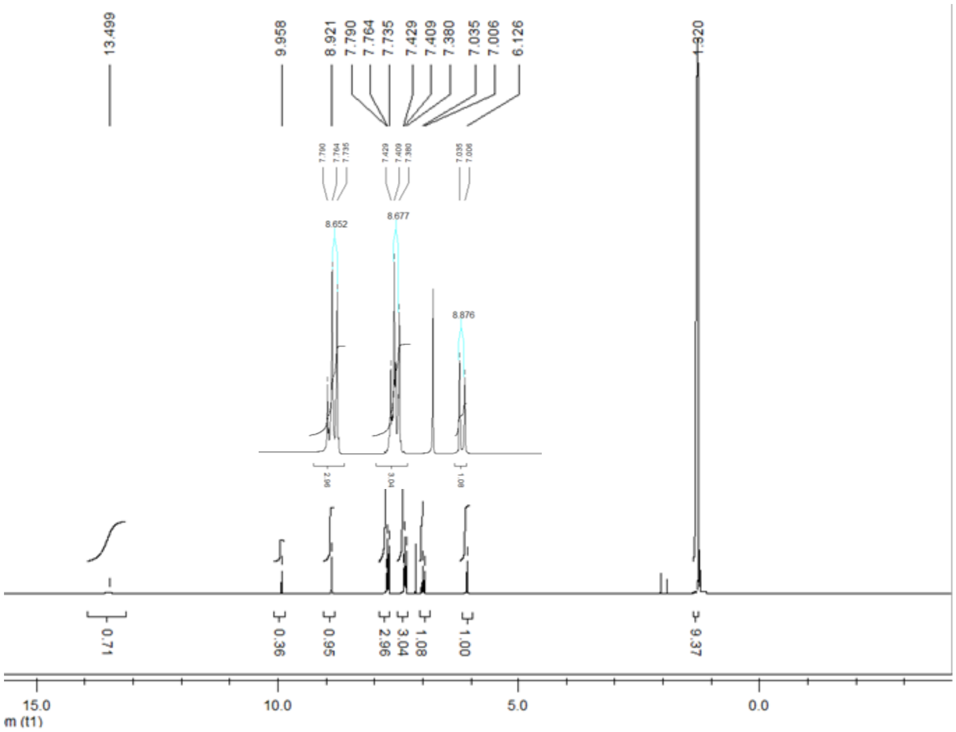

Figure S18. ^{1}H-NMR for compound **ClAzoNH** in $CDCl_3$.

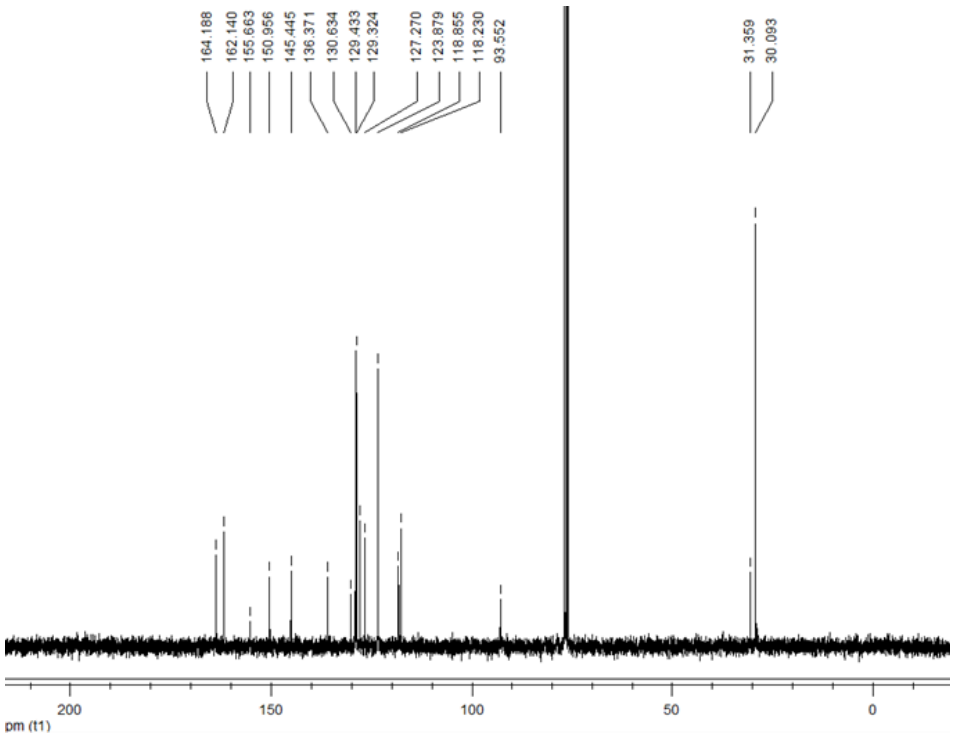

Figure S19. ^{13}C-NMR spectrum of **ClAzoNH** in $CDCl_3$.

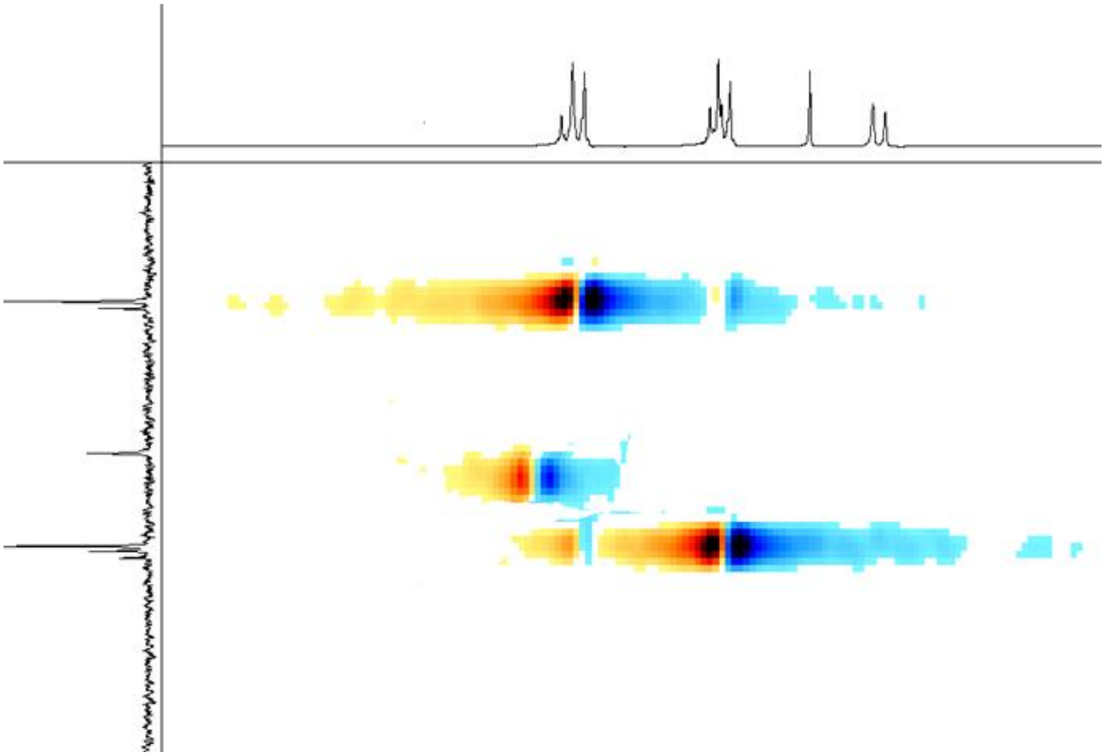

Figure S20. HSQC-NMR spectrum of **ClAzoNH** in $CDCl_3$.

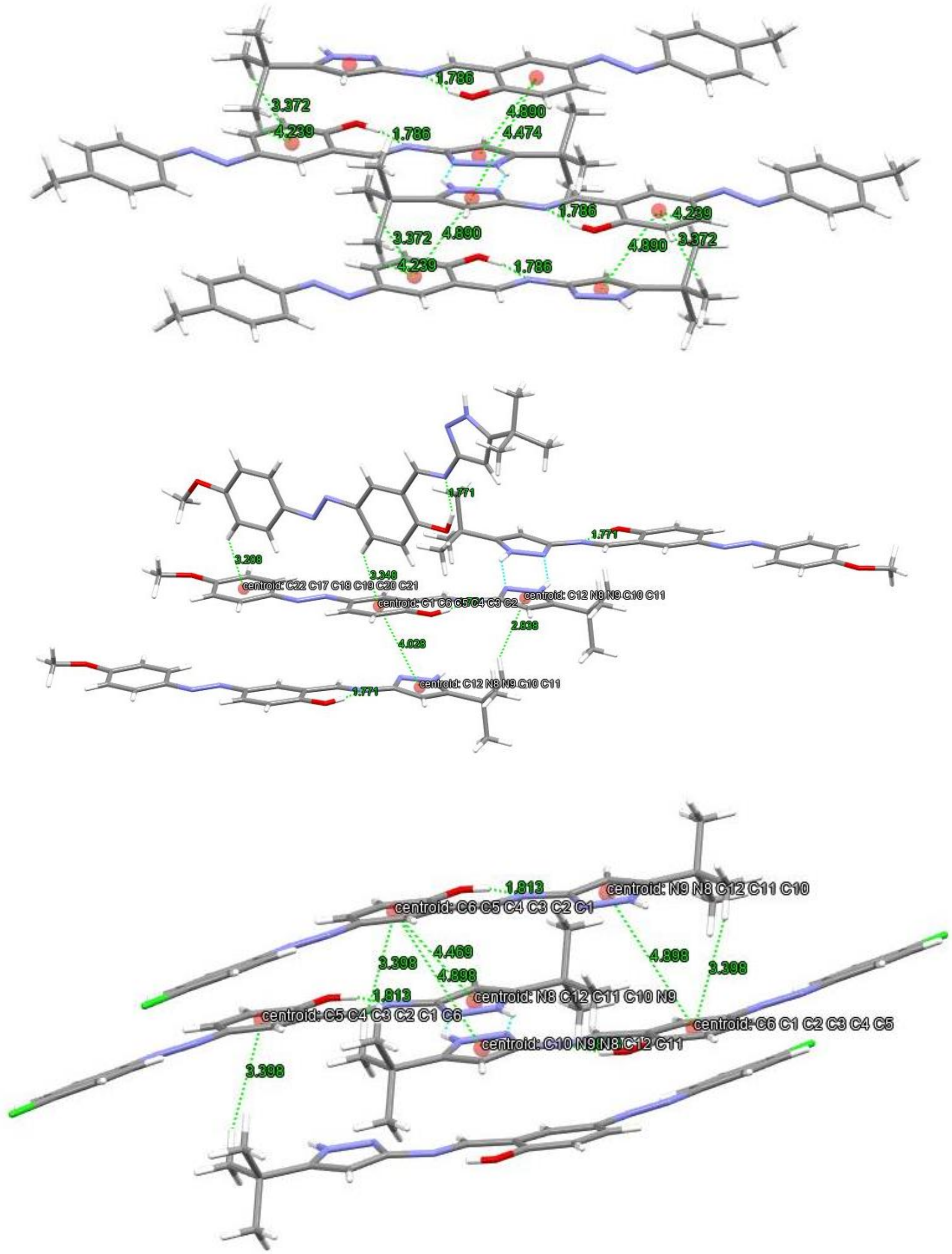

Figure S21. Centroid-centroid interaction distances in **MeAzoNH**, **MeOAzoNH,** and **ClAzoNH**.

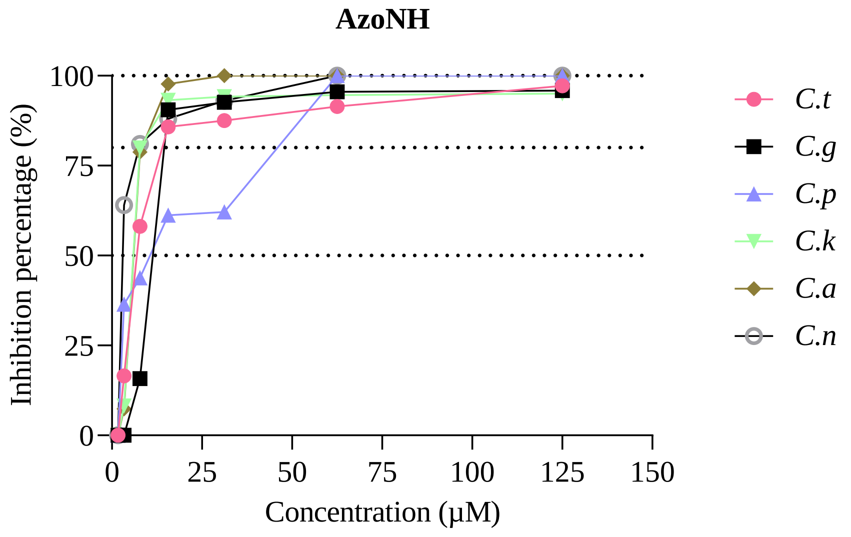

Figure S22. Percentage inhibition *versus* concentration of **AzoNH** on *Candida tropicalis* (*C.t*), *Candida glabrata* (*C.g*), *Candida parapsilosi* (*C.p*), *Candida krusei* (*C.k*), *C. albicans* (*C.a*) and *C. neoformans* (*C.n*).

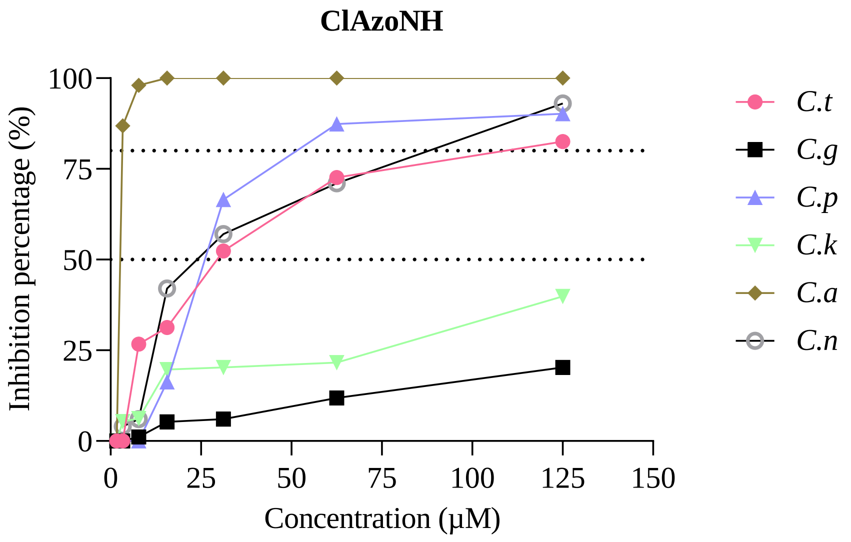

Figure S23. Percentage inhibition *versus* concentration of **ClAzoNH** on *Candida tropicalis* (*C.t*), *Candida glabrata* (*C.g*), *Candida parapsilosi* (*C.p*), *Candida krusei* (*C.k*), *C. albicans* (*C.a*) and *C. neoformans* (*C.n*).

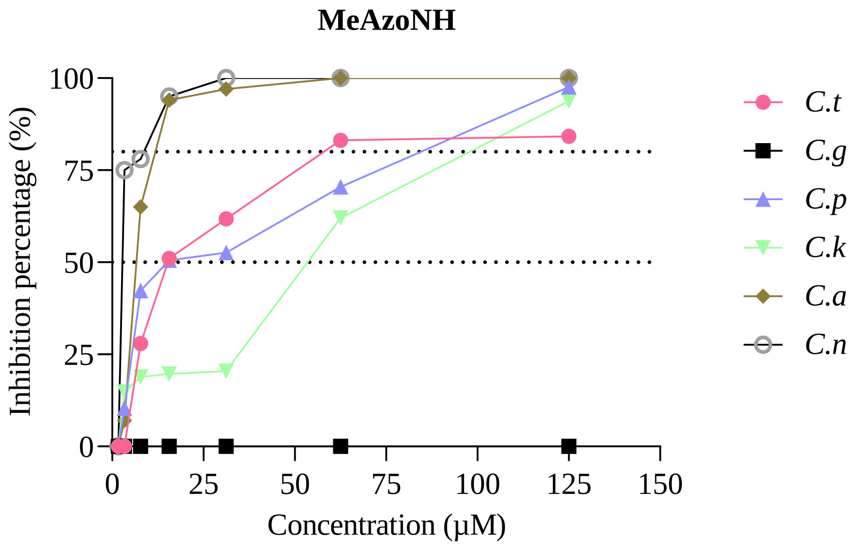

Figure S24. Percentage inhibition *versus* concentration of **MeAzoNH** on *Candida tropicalis* (*C.t*), *Candida glabrata* (*C.g*), *Candida parapsilosi* (*C.p*), *Candida krusei* (*C.k*), *C. albicans* (*C.a*) and *C. neoformans* (*C.n*).

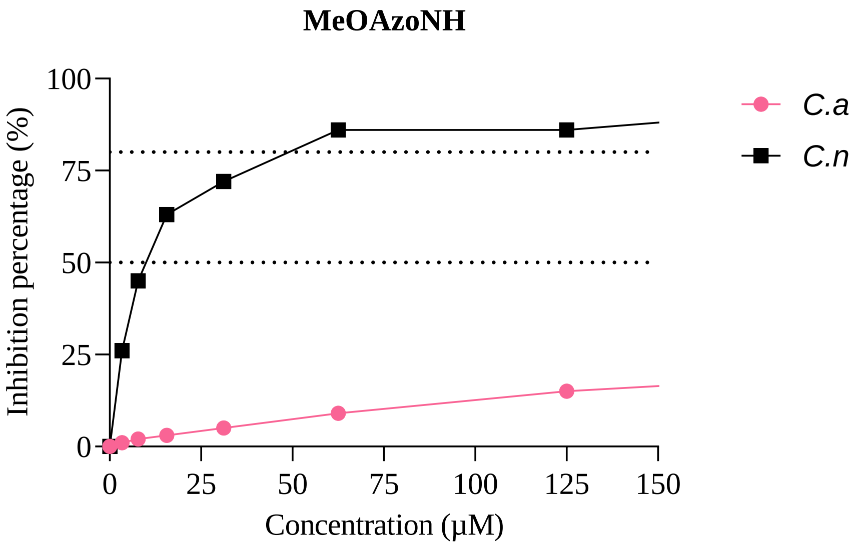

Figure S25. Percentage inhibition *versus* concentration of **MeOAzoNH** on *C. albicans* (*C.a*) and *C. neoformans* (*C.n*).

Table S1. Crystallographic data and refinement parameters for **AzoNH**, **MeAzoNH**, **MeOAzoNH**, and **ClAzoNH**.

**Compound**	**AzoNH**	**MeAzoNH**	**ClAzoNH**	**MeOAzoNH**
**Emp. Formula**	$C_{20}H_{21}N_5O$	$C_{21}H_{23}N_5O$	$C_{20}H_{20}ClN_5O$	$C_{21}H_{23}N_5O_2$
**FW (g/mol)**	347.42	361.44	381.86	377.44
**Temperature (K)**	298(2)	298(2)	298(2)	293(2)
**λ (Å)**	0.71073	0.71073	0.71073	0.71073
**Crystal system**	Triclinic	Triclinic	Triclinic	orthorhombic
**Space Group**	P-1	P-1	P-1	2ab
**a (Å)**	6.0906(4)	6.139(4)	6.133(4)	27.4083(9)
**b (Å)**	10.3193(7)	10.263(7)	10.246(6)	5.65030(10)
**c (Å)**	14.7276(11)	15.379(11)	15.353(10)	26.0273(9)
**α (Å)**	95.271(2)	90.48(2)	88.994(18)	90
**β (Å)**	90.302(3)	89.78(2)	89.891(17)	90
**γ (Å)**	98.116(2)	97.86(2)	82.530(18)	90
**Volume ($Å^3$)**	912.34(11)	959.8(12)	956.4(10)	4030.7(2)
**Z**	2	2	2	8
**ρ_{cal} (mg×m^3)**	1.265	1.251	1.326	1.244
**Abs. Coeff. (mm^{-1})**	0.082	0.081	0.220	0.083
**F(000)**	368	384	400	1600
**θ range (°)**	2.33-30.04	2.39-30.13	2.39-27.27	-
**Reflections collected/unique**	36671/5344	5755/5755	21751/4210	14681/ 4654
**[R(int)]**	[0.0633]	[0.1234]	[0.1702]	[0.1552]
**Completeness (%)**	99.6	98.8	99.8	99.3
**Data/restraints/parameters**	5344/0/244	5755/1/255	4210/0/253	4654/0/259
**GoF on F^2**	1.011	1.027	0.997	0.846
**R1 [I>2σ(I)]**	0.0572	0.0970	0.0703	0.0560
**wR2 [I>2σ(I)]**	0.1354	0.2389	0.2254	0.2370
